# ﻿Taxonomic review of the grasshopper genus *Pteropera* Karsch, 1891 (Orthoptera, Acrididea, Catantopinae) with description of three new species and a preliminary phylogeny of the Cameroonian species

**DOI:** 10.3897/zookeys.1216.130270

**Published:** 2024-10-25

**Authors:** Jeanne Agrippine Yetchom Fondjo, Armand Richard Nzoko Fiemapong, Maurice Tindo, Tarekegn Fite Duressa, Slobodan Ivković, Martin Husemann

**Affiliations:** 1 Zoology Unit, Laboratory of Biology and Physiology of Animal Organisms, Graduate School in Fundamental and Applied Sciences, University of Douala, Douala, Cameroon University of Douala Douala Cameroon; 2 Staatliches Museum für Naturkunde Karlsruhe, Karlsruhe, Germany Staatliches Museum für Naturkunde Karlsruhe Karlsruhe Germany; 3 University of Neuchâtel, Neuchâtel, Switzerland University of Neuchâtel Neuchâtel Switzerland; 4 School of Plant Sciences, College of Agriculture and Environmental Sciences, Haramaya University, Dire Dawa, Ethiopia Haramaya University Dire Dawa Ethiopia

**Keywords:** DNA barcodes, integrated taxonomy, short-horned grasshopper, tropical Africa

## Abstract

The Afrotropical grasshopper genus *Pteropera* Karsch, 1891, is reviewed. Some species present in Cameroon are described, *Pteroperaaugustini* Donskoff, 1981, is recorded for the first time in the country, and three new species are described from Cameroon, *Pteroperakennei* Yetchom & Husemann, **sp. nov.**, *Pteroperamatzkei* Yetchom & Husemann, **sp. nov.** and *Pteroperamissoupi* Yetchom & Husemann, **sp. nov.**, increasing the number of *Pteropera* species in Cameroon from eight to 12, and overall to 30 species in Central Africa. An updated key of *Pteropera* is provided. Photographs with data on the distributions of all known species are given. In addition, a phylogenetic tree was constructed using maximum likelihood and Bayesian inference on the basis of a concatenated dataset of COI, 16S, and 12S markers of available Cameroonian species. The maximum likelihood and Bayesian inference analyses of the concatenated datasets resulted in a well-resolved phylogeny of the group and species of *Pteropera* were recovered as monophyletic, largely with high support. In all cases, the discrimination of all studied species based on barcode information was congruent with the species limits determined by traditional taxonomy. Our findings show the potential of integrative taxonomy to resolve the relationships among grasshoppers below the family level. Further analyses, including more comprehensive taxon sampling and additional nuclear markers, are needed, and the occurrence of several taxa still needs to be confirmed in African rainforests.

## ﻿Introduction

*Pteropera* Karsch, 1891, is a micropterous Afrotropical grasshopper genus belonging to the subfamily Catantopinae. This flightless grasshopper genus is morphologically similar to its close relative *Serpusia* Karsch (Johnston 1956; Rowell et al. 2018). Species of the genus are common in forests, at forest edges, and in agrosystems, and most of them have restricted distribution ranges.

*Pteropera* was originally described for a single species, *Pteroperaverrucigena* Karsch, and remained monotypic until the description of *P.pictipes* by Bolívar in 1908. Moreover, *P.karschi* (Bolívar, 1905), previously included in the genus *Aresceutica* Karsch, 1896, was included in the genus *Pteropera* by Donskoff (1981). After 12 years, *P.uniformis* Bruner, 1920 was described. Shortly thereafter, Ramme (1929) revised the genus on the basis of external morphology, described two additional species (*P.carnapi* Ramme, 1929, *P.zenkeri* Ramme, 1929), and transferred *P.spleniata* (Karsch, 1896) and *P.femorata* (Giglio-Tos, 1907), originally placed in the genus *Serpusia*, into the genus *Pteropera*. Moreover, Ramme (1929) proposed two keys to *Pteropera* species (one based on males and one on females), which included eight species distinguished on the basis of external morphological features and coloration. Thereafter, Ramme (1929) indicated *P.pictipes* as synonyms of *P.femorata*. Fifty-two years later, Donskoff (1981) conducted a complete revision of the genus on the basis of external morphology, coloration, and features of the genitalia and described 21 new species; by then, the genus comprised 27 valid species described from Central African forests only. Since, no further taxonomic work dedicated to this genus has been done. Given the large areas where no inventory works have been conducted thus far, it is likely that *Pteropera* is more diverse than currently known. Furthermore, to date, no molecular data for this genus are available. Thus, the main objective of this work is to shed light on the taxonomic status of grasshopper species of the genus *Pteropera* through an integrative approach, including morphometric, morphological, and molecular analyses. Herein, a description of three species new to science, distribution maps, an updated key to species, an annotated list, and photographs of all species of *Pteropera*, and the first phylogenetic tree for Cameroonian species are provided.

## ﻿Materials and methods

### ﻿Specimen collection and morphological studies

Field surveys were conducted from June 2017 to April 2022 at various locations situated in the central (Ongot), eastern (Somalomo, Dja), and littoral (Sohock, Koukoué, Iboti) regions of Cameroon. The grasshopper samples were collected using sweep nets and hand catches.

Specimens were identified using the identification key of Donskoff (1981). A total of six species of *Pteropera*, including three species new to science in the study area, were identified. In addition, type specimens held in the Muséum Nationale d´Histoire Naturelle Paris, France (**MNHN**) and the Museum für Naturkunde Berlin, Germany (**MfN**) were examined. Fresh samples were stored in absolute ethanol for further DNA analysis. They are kept as vouchers in the entomological collection at the Staatliches Museum für Naturkunde Karlsruhe, Germany (**SMNK**).

To study male genitalia, the standard methods of Kevan et al. (1969) and Martinelli et al. (2017) for the extraction and preparation of internal genitalia were followed. The genitalia were extracted from the grasshopper body using finely hooked forceps. The extracted internal genitalia were placed in a 1.5 mL microcentrifuge tube containing a solution of 5 µL of proteinase K (20 mg/mL) and 25 µL of buffer (pH 8.0, 10 mM Tris-Cl, 25 mM EDTA, 100 mM NaCl, 0.5% SDS) and were incubated overnight in an incubator at 55 °C. The next day, the genitalia were gently separated from the digestion solution and then kept at 95 °C for 10 min to inactivate the enzyme; the preparations were then washed with double distilled water (ddH_2_O). The terminology of male genitalia and female spermatheca follows Donskoff (1981) and Rowell (2013).

Photographs of the habitus of types and allotypes held by the MNHN were captured with a Nikon D60 digital camera. Photographs of some samples were taken at the Zoologisches Museum Hamburg, Germany (**ZMH**) with a high-resolution DUN Inc. stacking system (DUN Inc. California, USA). Images of male and female genitalia were also taken at the ZMH with a Keyence VHX-7000 digital microscope (London, UK).

Measurements were obtained using a digital caliper (at a scale of 0.01 mm). All the measurements are given in millimeters (mm). For all traits, male and female samples were measured separately. For each sample, the following measurements were taken: **HeadL**: length of the head; **HeadW**: width of the head; **AntenL**: length of the antenna; **I.O.D.**: interocular distance; **FastigL**: length of the fastigium of vertex; **PronotL**: length of the pronotum in the midline; **PronotW**: pronotum width; **TegL**: length of the tegmina; **TL**: hind tibia length; **FL**: maximum length of the hind femur; **FW**: width of the hind femur, measured as the distance between the two parallel lines running through the dorsal and ventral extremities of the femur, drawn parallel to the long axis of the femur; and **BodyL**: body length, measured from the tip of the front to the hindmost tip of the abdomen. The measurements of the samples (Table 1) correspond to the average value of the different body parts of the grasshoppers plus the standard deviation (SD).

**Table 1.** Measurements in millimeters (mm) of the examined *Pteropera* species currently known from Cameroon.

Species	*Pteroperacarnapi* Ramme, 1929		*Pteroperadescampsi* Donskoff, 1981		*Pteroperakarschizenkeri* Ramme, 1929		*Pteroperakennei* sp. nov.	
Parameters	Male	Female	Male	Female	Male	Female	Male	Female
**HeadL**	1.95 ± 0.15 (*n* = 7)	2.34 ± 0.18 (*n* = 6)	2.04 ± 0.20 (*n* = 8)	2.19 ± 0.13 (*n* = 8)	4.54 ± 1.04 (*n* = 4)	4.54 ± 1.04 (*n* = 4)	2.77 ± 0.24 (*n* = 20)	3.07 ± 0.11 (*n* = 5)
**HeadW**	3.08 ± 0.12 (*n* = 7)	3.67 ± 0.07 (*n* = 6)	2.99 ± 0.10 (*n* = 8)	3.61 ± 0.20 (*n* = 8)	3.15 ± 0.13 (*n* = 4)	3.15 ± 0.13 (*n* = 4)	3.05 ± 0.15 (*n* = 20)	3.73 ± 0.23 (*n* = 5)
**AntenL**	9.57 ± 0.73 (*n* = 6)	10.34 ± 0.62 (*n* = 6)	9.64 ± 0.66 (*n* = 7)	10.22 ± 0.69 (*n* = 7)	11.62 ± 0.50 (*n* = 4)	11.62 ± 0.50 (*n* = 4)	9.73 ± 0.37 (*n* = 20)	10.36 ± 0.65 (*n* = 5)
**I.O.D.**	0.47 ± 0.19 (*n* = 7)	0.60 ± 0.16 (*n* = 6)	0.64 ± 0.08 (*n* = 8)	0.64 ± 0.08 (*n* = 8)	0.37 ± 0.12 (*n* = 4)	0.37 ± 0.12 (*n* = 4)	0.55 ± 0.12 (*n* = 20)	0.68 ± 0.03 (*n* = 5)
**PronotL**	4.32 ± 0.25 (*n* = 7)	5.02 ± 0.13 (*n* = 6)	5.06 ± 0.32 (*n* = 8)	5.06 ± 0.32 (*n* = 8)	4.27 ± 0.07 (*n* = 4)	4.27 ± 0.07 (*n* = 4)	4.01 ± 0.26 (*n* = 20)	4.94 ± 0.35 (*n* = 5)
**PronotW**	3.54 ± 0.38 (*n* = 7)	4.38 ± 0.24 (*n* = 6)	4.17 ± 0.26 (*n* = 8)	4.17 ± 0.26 (*n* = 8)	3.56 ± 0.26 (*n* = 4)	3.56 ± 0.26 (*n* = 4)	3.37 ± 0.28 (*n* = 20)	4.39 ± 0.28 (*n* = 5)
**TegL**	3.58 ± 0.19 (*n* = 7)	4.43 ± 0.47 (*n* = 6)	3.73 ± 0.41 (*n* = 7)	4.31 ± 0.63 (*n* = 7)	4.17 ± 0.33 (*n* = 4)	4.17 ± 0.33 (*n* = 4)	3.80 ± 0.29 (*n* = 20)	4.26 ± 0.56 (*n* = 5)
**TL**	10.45 ± 0.15 (*n* = 7)	12.42 ± 0.71 (*n* = 6)	12.11 ± 0.55 (*n* = 8)	12.11 ± 0.55 (*n* = 8)	11.59 ± 0.19 (*n* = 4)	11.59 ± 0.19 (*n* = 4)	10.27 ± 0.43 (*n* = 20)	12.49 ± 0.57 (*n* = 5)
**FL**	12.07 ± 0.25 (*n* = 7)	14.39 ± 0.48 (*n* = 5)	12.17 ± 0.54 (*n* = 7)	14.03 ± 0.82 (*n* = 7)	13.52 ± 0.04 (*n* = 4)	13.52 ± 0.04 (*n* = 4)	11.91 ± 0.35 (*n* = 20)	14.32 ± 0.56 (*n* = 5)
**FW**	1.61 ± 0.21 (*n* = 7)	1.91 ± 0.11 (*n* = 5)	1.64 ± 0.22 (*n* = 7)	1.75 ± 0.22 (*n* = 7)	3.10 ± 0.21 (*n* = 4)	3.10 ± 0.21 (*n* = 4)	3.10 ± 0.15 (*n* = 20)	3.67 ± 0.23 (*n* = 5)
**BodyL**	19.92 ± 1.26 (*n* = 7)	25.35 ± 2.20 (*n* = 6)	24.52 ± 2.41 (*n* = 8)	24.52 ± 2.41 (*n* = 8)	21.24 ± 0.58 (*n* = 4)	21.24 ± 0.58 (*n* = 4)	18.15 ± 1.71 (*n* = 20)	23.08 ± 1.69 (*n* = 5)
**Species**	***Pteroperamatzkei* sp. nov.**		***Pteroperamissoupi* sp. nov.**		***Pteroperauniformis* Bruner, 1920**		***Pteroperaverrucigena* Karsch, 1891**	
**Parameters**	Male	Female	Male	Female	Male	Female	Male	Female
**HeadL**	4.54 ± 1.04 (*n* = 4)	5.33 ± 1.85 (*n* = 2)	2.41 ± 0.53 (*n* = 8)	2.87 ± 0.56 (*n* = 7)	2.16 ± 0.00 (*n* = 1)	–	2.04 ± 0.24 (*n* = 2)	2.24 (*n* = 1)
**HeadW**	3.15 ± 0.13 (*n* = 4)	3.46 ± 0.71 (*n* = 2)	3.29 ± 0.26 (*n* = 8)	3.64 ± 0.69 (*n* = 7)	3.10 ± 0.00 (*n* = 1)	–	3.43 ± 0.21 (*n* = 2)	4.48 (*n* = 1)
**AntenL**	11.62 ± 0.50 (*n* = 4)	12.75 ± 0.54 (*n* = 2)	10.07 ± 0.57 (*n* = 8)	10.71 ± 0.33 (*n* = 7)	10.99 ± 0.00 (*n* = 1)	–	–	–
**I.O.D.**	0.37 ± 0.12 (*n* = 4)	0.69 ± 0.68 (*n* = 2)	0.55 ± 0.08 (*n* = 8)	0.77 ± 0.19 (*n* = 7)	0.44 ± 0.00 (*n* = 1)	–	0.66 ± 0.30 (*n* = 2)	0.79 (*n* = 1)
**PronotL**	4.27 ± 0.07 (*n* = 4)	5.47 ± 0.11 (*n* = 2)	4.50 ± 0.26 (*n* = 8)	5.35 ± 0.16 (*n* = 7)	4.58 ± 0.00 (*n* = 1)	–	4.46 ± 0.45 (*n* = 2)	5.58 (*n* = 1)
**PronotW**	3.56 ± 0.26 (*n* = 4)	4.74 ± 0.01 (*n* = 2)	3.81 ± 0.40 (*n* = 8)	4.52 ± 0.56 (*n* = 7)	3.62 ± 0.00 (*n* = 1)	–	3.74 ± 0.32 (*n* = 2)	4.89 (*n* = 1)
**TegL**	4.17 ± 0.33 (*n* = 4)	5.53 ± 0.81 (*n* = 2)	3.81 ± 0.43 (*n* = 7)	4.79 ± 0.40 (*n* = 7)	3.76 ± 0.00 (*n* = 1)	–	3.70 ± 0.28 (*n* = 2)	5.91 (*n* = 1)
**TL**	11.59 ± 0.19 (*n* = 4)	14.28 ± 0.33 (*n* = 2)	11.29 ± 0.28 (*n* = 8)	13.99 ± 0.46 (*n* = 6)	10.64 ± 0.00 (*n* = 1)	–	11.30 ± 0.80 (*n* = 2)	14.72 (*n* = 1)
**FL**	13.52 ± 0.04 (*n* = 4)	16.40 ± 0.69 (*n* = 2)	13.20 ± 0.39 (*n* = 8)	16.32 ± 0.71 (*n* = 7)	12.83 ± 0.00 (*n* = 1)	–	12.84 ± 0.78 (*n* = 2)	17.23 (*n* = 1)
**FW**	3.10 ± 0.21 (*n* = 4)	3.73 ± 0.01 (*n* = 2)	1.65 ± 0.16 (*n* = 8)	1.98 ± 0.10 (*n* = 7)	1.75 ± 0.00 (*n* = 1)	–	1.74 ± 0.11 (*n* = 2)	1.69 (*n* = 1)
**BodyL**	21.24 ± 0.58 (*n* = 4)	27.65 ± 0.57 (*n* = 2)	21.02 ± 1.51 (*n* = 7)	26.38 ± 1.35 (*n* = 7)	19.88 ± 0.00 (*n* = 1)	–	20.37 ± 0.43 (*n* = 2)	28.86 (*n* = 1)

The measurements represent the average value of the different body parts plus the standard deviation. **HeadL**: length of head; **HeadW**: width of head; **AntenL**: length of antenna; **I.O.D.**: interocular distance; **FastigL**: length of fastigium of vertex; **PronotL**: Length of the pronotum in the midline; **PronotW**: pronotum width; **TegL**: length of tegmina; **TL**: hind tibia length; **FL**: maximum length of the hind femur; **FW**: width of hind femur, measured as the distance between the two parallel lines running through the dorsal and ventral extremities of the femur, drawn parallel to the long axis of the femur; **BodyL**: body length, measured from the tip of the frons to the hindmost tip of the abdomen; **n**: number of measured individuals; -: not applicable.

Distributional data were obtained from geographical coordinates recorded during field observations and from locality records taken from specimen labels in the ZMH and the MNHN collections. The distribution maps of all the species were generated via QGIS 3.28.3.

### ﻿Depositories

**ANSP** Academy of Natural Science of Philadelphia

**EMT** Egyptian Museum of Turin

**MfN** Museum für Naturkunde Berlin, Germany

**MNHN** Muséum National d´Histoire Naturelle Paris, France

**RMCA** Royal Museum for Central Africa Tervuren, Belgium

**RBINS** Royal. Belgian Institute of Natural Sciences

**SMNK** Staatliches Museum für Naturkunde Karlsruhe, Germany

**ZMH** Zoologisches Museum Hamburg, Germany

### ﻿DNA extraction, PCR amplification, and sequencing

To perform molecular analyses, genomic DNA was extracted from the femoral muscle tissue of 41 *Pteropera* specimens and 11 outgroups samples (Table 3) stored in 96% ethanol at the ZMH. DNA was isolated using a high-salt extraction protocol (Paxton et al. 1996). To amplify the nucleotide sequences of the grasshopper COI, 16S and 12S markers, the primer pairs LCO (5´-GTCAACAAATCATAAAGATATTGG) and HCO (5´-AAACTTCAGGGTGACCAAAAAATCA) (Folmer et al. 1994), 16S-F (5´-CGCCTGTTTAACAAAAACAT) and 16S-R (5´-CCGGTCTGAACTCAGATCACGT) (Palumbi et al. 1991), 12S-F (5´-AAACTAGGATTAGATACCCTATTAT) and 12S-R (5´-AAGAGCGACGGGCGATGTGT) (Bruvo-Madrić et al. 2005) were used.

The master mix contained 10.78 µL of nuclease-free H_2_O, 1.5 µL of DreamTaq Buffer 10× (Thermo Fischer Scientific, Waltham, Massachusetts), 0.75 µL of the respective forward and reverse primers, 0.12 µL of dNTPs (VWR International, Radnor, Pennsylvania), 0.2 µL of DreamTaq DNA Polymerase (Thermo Fischer Scientific, Waltham, Massachusetts), and 1 µL of the template.

The PCR profile for the COI gene consisted of an initial denaturation step of 3 min at 94 °C, followed by 35 cycles of 30 s at 94 °C, an annealing step of 45 s at 50 °C, an extension step of 1 min at 72 °C, with a final extension of 10 min at 72 °C.

The PCR profile for the 16S gene consisted of an initial denaturation step of 3 min at 95 °C, followed by 35 cycles of 30 s at 95 °C, an annealing step of 45 s at 61 °C, an extension step of 1 min at 72 °C, with a final extension of 10 min at 72 °C.

The PCR profile for the 12S gene consisted of an initial denaturation step of 3 min at 95 °C, followed by 35 cycles of 30 s at 95 °C, an annealing step of 30 s at 60 °C, an extension step of 1 min at 72 °C, with a final extension of 5 min at 72 °C.

The PCR amplicons were checked on 1% agarose gels stained with GelRed (Biotium, Remont, CA, USA). Successfully amplified samples were purified with an ExoSap Enzyme cocktail (VWR, Pennsylvania, USA). The purified PCR products were then sequenced in both directions by Macrogen Europe (Amsterdam, Netherlands).

The newly obtained sequences were deposited in GenBank under the accession numbers indicated in Table 3.

For our study, the outgroups were selected among representatives from a few genera of the subfamily Catantopinae, on the basis of their close relationship with the ingroup. These include *Catantopsstramineus* (Walker, 1870), *Exopropacrismodica* (Karsch, 1893) and *Parapropacrisnotatus* (Karsch, 1891). In addition, we successfully sequenced the 16S fragment from 43 samples including 35 *Pteropera* samples and 8 samples from outgroups. For the 12S fragment, we sequenced 38 samples, including 34 *Pteropera* samples and four samples from outgroups.

The DNA sequences were edited using the BioEdit Sequence Alignment Editor v. 7.7.1 (Hall 1999). The sequences were further assembled and aligned in Geneious Pro (Kearse et al. 2012) using the MUSCLE algorithm (Edgar 2004). The aligned sequences were further visualized in SeaView (https://doua.prabi.fr/software/seaview). We checked for pseudogenes (numts) by translating sequences into amino acids using the invertebrate mitochondrial code and checking for frameshifts. Furthermore, NCBI BLAST databases were used to check for species identity and hence any contamination.

With our molecular datasets, we conducted one multilocus analysis (COI_16S_12S) with maximum likelihood (ML) and Bayesian inference (BI) methods. Phylogenetic tree based on the maximum likelihood (ML) method was reconstructed using the IQ-Tree software v. 1.6.12 (Nguyen et al. 2015). A bootstrap analysis was performed with 1000 replicates. The Bayesian analyses were performed using MrBAYES v. 3.2.7a (Ronquist et al. 2012b). Analyses were run for 1 million generations, with sampling every 100 generations for a total of 10,000 trees. The first 25% of the samples were discarded as burn-in. The average split frequencies were less than 0.01, indicating convergence of the analyses. The final trees were visualized with FigTree v. 1.4.2 (https://github.com/rambaut/figtree/releases; Rambaut 2010).

## ﻿Results

### ﻿Phylogenetic analysis

**Figure 1.**
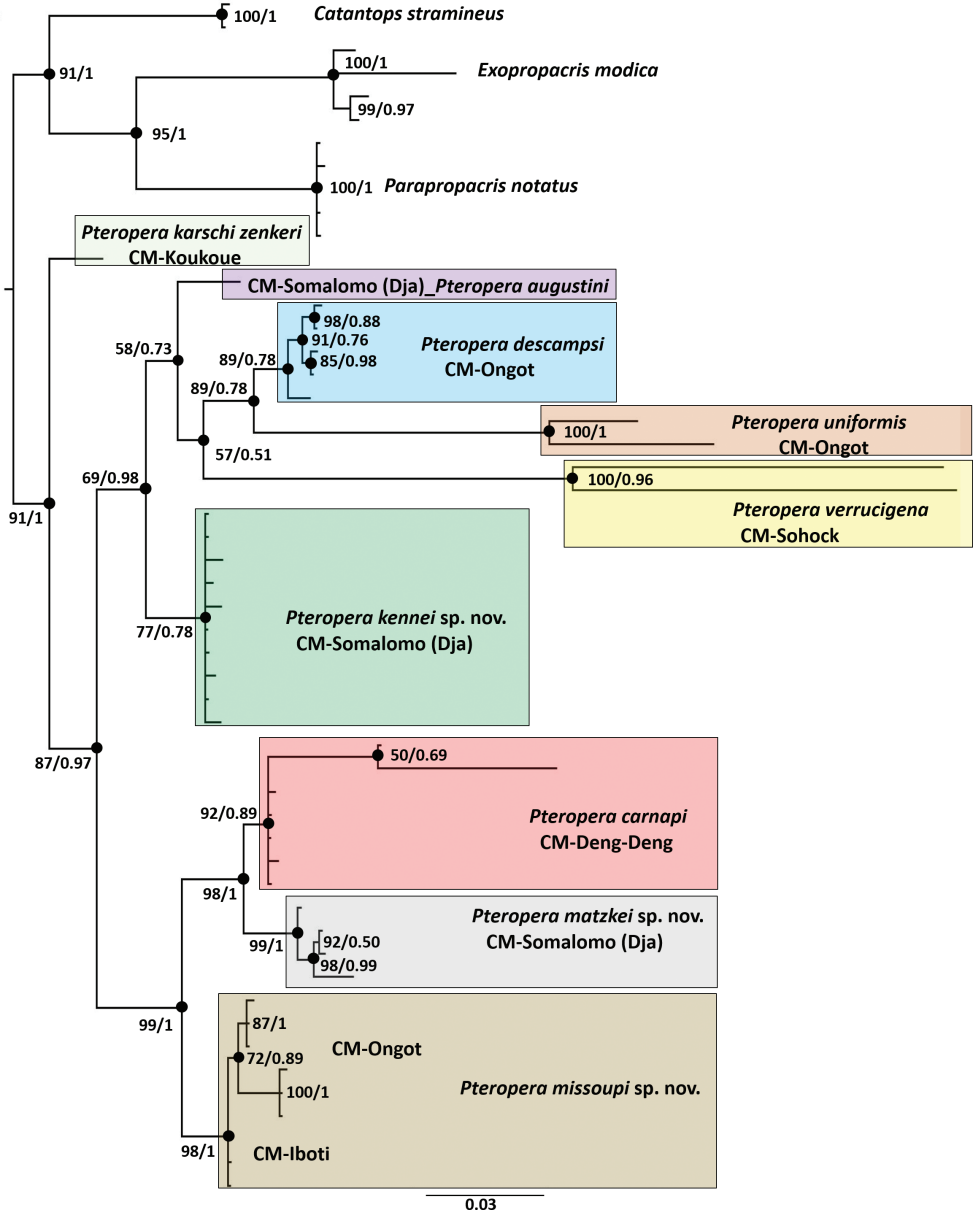
Phylogenetic tree built from the maximum likelihood (ML) and Bayesian inference (BI) analyses of the concatenated (COI/16S/12S) dataset. The numbers close to the nodes of the tree are the bootstrap support (%) and the Bayesian posterior probabilities (PP). The collection localities are also indicated preceded by CM (Cameroon).

The COI fragment was sequenced from 35 *Pteropera* specimens and 11 outgroup sample (Table 3). After trimming, the final alignment of the COI marker comprised 658 bp for 46 sequences of 11 taxa (including outgroups). In total, 43 sequences of the 16S from ten species (including outgroups), each 510 bp, were analyzed. Three outgroup sequences were missing compared with those in the COI datasets. The analyses of 16S alone yielded an unresolved tree. We analyzed a total of 352 bp for 37 sequences of 12 taxa (including outgroups) in the 12S region. The 12S topologies within *Pteropera* based on both ML and BI also resulted in largely unresolved trees. We concatenated all the loci and constructed two phylogenetic trees (one with ML and one with BI), on the basis of the concatenated sequence alignments of the three individual gene datasets (COI = 658 bp, 16S = 510 bp, 12S = 352 bp). The concatenated sequence alignment had a length of 1520 bp. Fig. 1 shows the combined majority-rule consensus tree obtained through the maximum likelihood and Bayesian inference analyses of the concatenated dataset. We recovered all included species of *Pteropera* as monophyletic, with high bootstrap values and posterior probabilities (Fig. 1). The ML and BI analyses of the combined dataset revealed a similar topology. In both the ML and BI datasets, the nine *Pteropera* species included in this work were grouped into five clades: (1) clade 1, containing *P.karschizenkeri*; (2) clade 2, containing *P.augustini* + *P.descampsi* + *P.uniformis* + *P.verrucigena*; (3) clade 3, containing *P.kennei* sp. nov.; (4) clade 4, containing *P.carnapi* + *P.matzkei* sp. nov.; and (5) clade 5, containing *P.missoupi* sp. nov. In addition, the basal taxon to the remaining *Pteropera* species was recovered by *P.karschizenkeri* with high support (PP > 0.95). Although the subclade formed by *P.carnapi* was reconstructed with ML analyses with high support, this was not supported by BI analyses (92% in ML; PP = 0.89 in BI analyses). In general, the delimitation of taxonomic units on the basis of genetic analyses was in line with the species limits obtained with traditional taxonomy.

### ﻿Taxonomic account

**Family Acrididae MacLeay, 1821**

**Subfamily Catantopinae Brunner von Wattenwyl, 1893**

**Genus group Serpusiae Johnston, 1956**

#### ﻿Genus Pteropera

Taxon classificationAnimaliaOrthopteraAcrididae

Karsch, 1891

E9868821-AC9F-5D83-B608-7D56C8F4A79B

Pteropera Karsch, 1891: 185 (type species: Pteroperaverrucigena Karsch, 1891, by original monotypy); Kirby 1910: 473; Ramme 1929: 358–360; Johnston 1956: 291; Sjöstedt 1931: 28–29; Johnston 1968: 239; Dirsh 1965: 338–339; Donskoff 1981: 33–88; Otte 1995: 331–333; Yetchom Fondjo et al. 2019: 317.

##### Diagnosis.

Of medium size (22.5 mm; 30.0 mm); integument moderately rugous dorsally and smooth ventrally; body and legs with inconspicuous hairs; antennal organ on the fifth segment before the apex; frons oblique (~ 45°); frontal ridge slightly curved, depressed near the median ocelli, with parallel carinae; fastigium of vertex short, triangular to hexagonal, more or less elongated, with the upper area very small, almost flat above; interocular distance narrower than or equal to the antennal scape; eyes large, globular, oval in profile, bean-shaped in dorsal view; ocelli large; pronotum cylindrical in cross-section at the typical groove, crossed by three transverse furrows; median carina faintly visible, lateral carina absent; metazona twice shorter than the prozona; anterior margin always notched, posterior margin excurved or notched; prosternal tubercle subconical, prominent, elevated, isolated; mesosternal lobes rounded. Tegmina lobiform, 3× longer than its width, covering the larger tympanum; wings less developed. Last article of the anterior and medial tarsi longer than the other two combined; Hind femur longer than wide; chevrons continuous and rounded in the outer median area; upper carinae serrate; upper basal lobe larger than the lower; hind tibia shorter than the femur, slightly S-curved, external apical spine absent, 8–10 spines on each upper margin; last tarsal segment as long as the other two combined; arolium larger and longer than the spurs. Supra-anal plate triangular, elongated; cerci slightly curved, conical, acute or truncated, sometimes with internal preapical lobules; male subgenital plate short, conical, or truncated; valves of ovipositor narrow, with curved apices, lower valves with small or no lateral projection; male genitalia: epiphallus bridge-shaped; bridge usually short, straight or arched, curved forward, reinforced in the vertical plane by a tubercle-like thickening and as prominent downwards as the lateral plates; ancorae short; lophi plate-shaped, aligned or forming an angle greater than 70°, posterior process not very prominent; oval sclerites small, rounded to subtriangular; cingulum horseshoe-shaped; rami of the cingulum not curved ventrally; ectophallus with two lower and two upper spiculated sheaths; intromission organ of aedeagus having four sclerotized blades and two upper spiculated sheaths; lower valves typically shorter than upper ones.

### ﻿List of *Pteropera* species known from African rainforests

**Figure 2.**
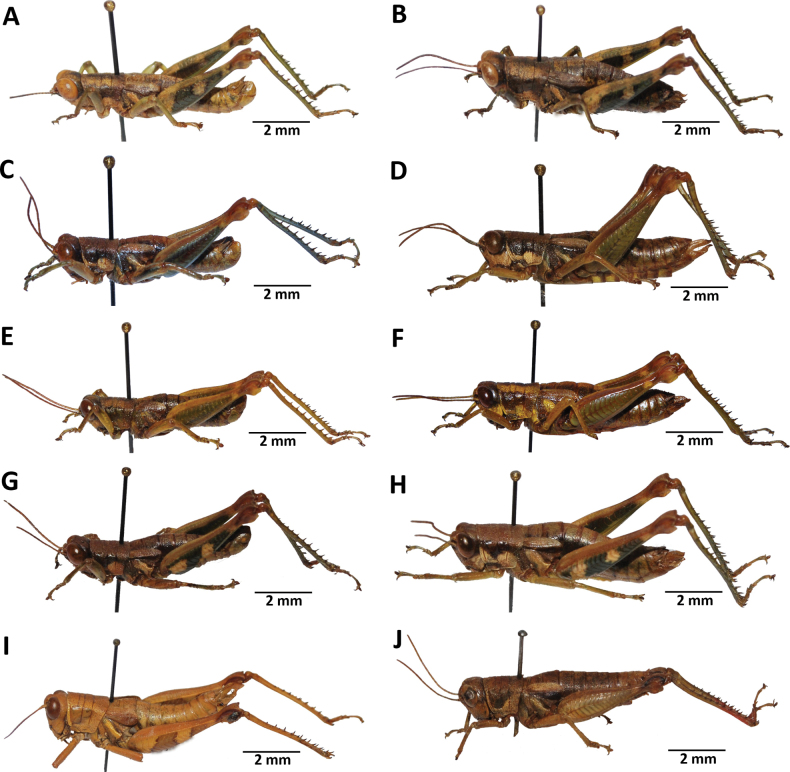
Images of holotypes, allotypes, and paratypes of *Pteropera* species in lateral view **A***P.augustini* (holotype ♂) **B***P.augustini* (allotype ♀) **C***P.balachowskyi* (holotype ♂) **D***P.balachowskyi* (allotype ♀) **E***P.bertii* (holotype ♂) **F***P.bertii* (paratype ♀) **G***P.brosseti* (holotype ♂) **H***P.brosseti* (allotype ♀) **I***P.basilewskyi* (paratype ♀) **J***P.bredoi* (holotype ♀).

**Figure 3.**
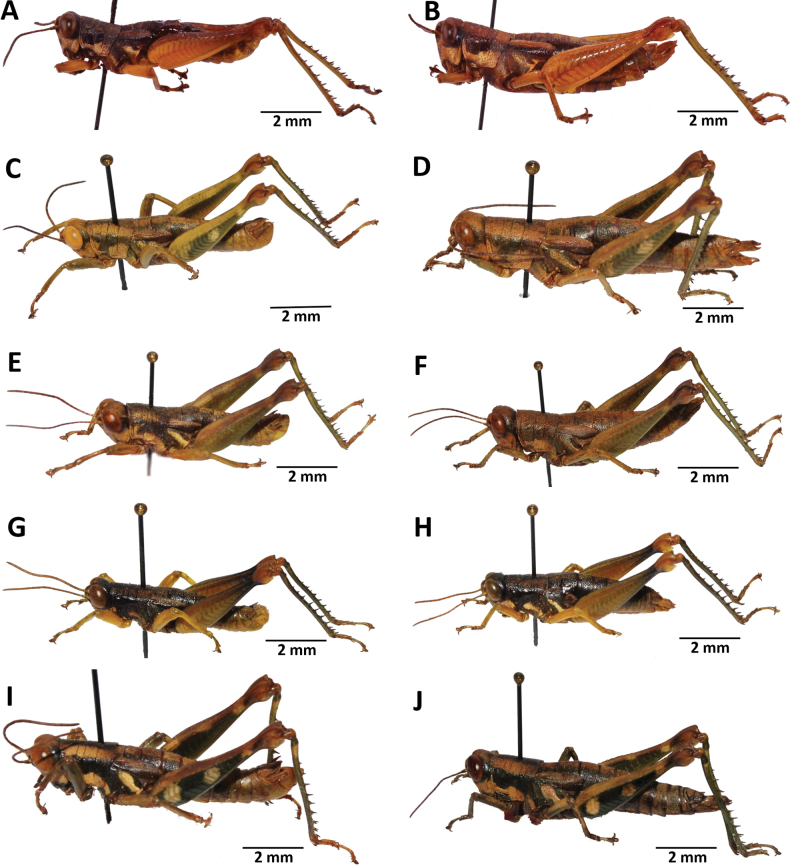
Images of holotypes and allotypes of *Pteropera* species in lateral view **A***P.carnapi* (holotype ♂) **B***P.carnapi* (paratype ♀) **C***P.congoensis* (holotype ♂) **D***P.congoensis* (allotype ♀) **E***P.cornici* (holotype ♂) **F***P.cornici* (allotype ♀) **G***P.descampsi* (holotype ♂) **H***P.descampsi* (allotype ♀) **I***P.descarpentriesi* (holotype ♂) **J***P.descarpentriesi* (allotype ♀).

**Figure 4.**
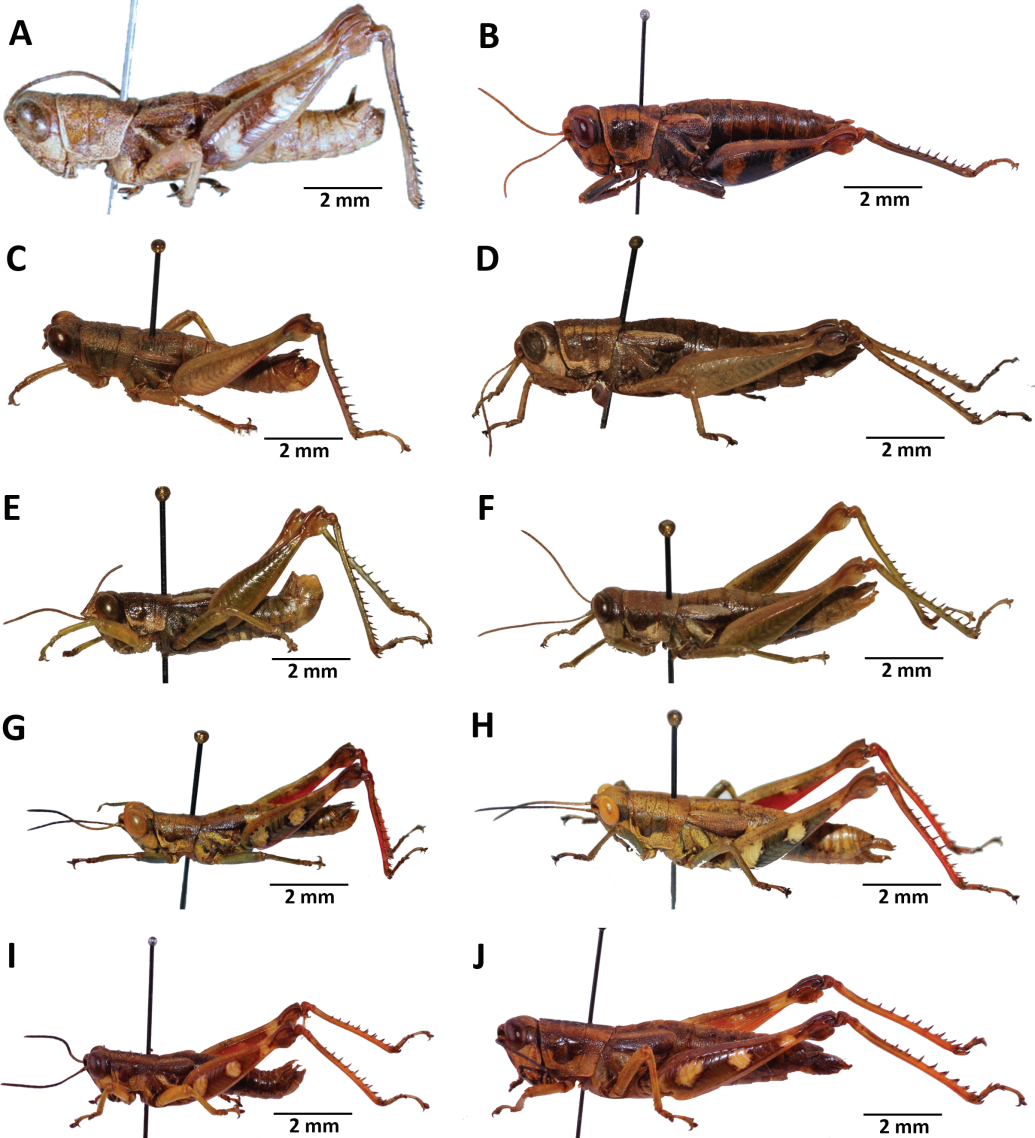
Images of holotypes, allotypes and paratypes of *Pteropera* species and subspecies in lateral view **A***P.femorata* (holotype ♂; Cigliano et al. 2024) **B***P.femorata* (♀) **C***P.grilloti* (holotype ♂) **D***P.meridionalis* (holotype ♀) **E***P.jeanninae* (holotype ♂) **F***P.jeanninae* (allotype ♀) **G***P.karschikarschi* (♂) **H***P.karschikarschi* (♀) **I***P.karschizenkeri* (holotype ♂) **J***P.karschizenkeri* (allotype ♀).

**Figure 5.**
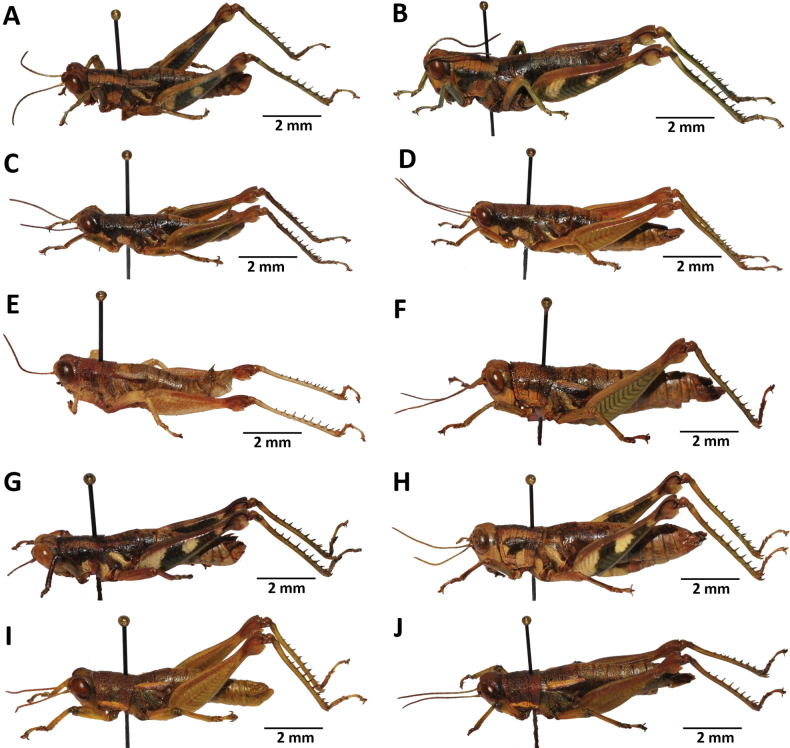
Images of Holotypes and Allotypes of *Pteropera* species in lateral view **A***P.menieri* (Holotype ♂) **B***P.menieri* (Allotype ♀) **C***P.mirei* (Holotype ♂) **D***P.mirei* (Allotype ♀) **E***P.morini* (Holotype ♂) **F***P.morini* (Allotype ♀) **G***P.pillaulti* (Holotype ♂) **H***P.pillaulti* (Allotype ♀) **I***P.poirieri* (Holotype ♂) **J***P.poirieri* (Allotype ♀).

**Figure 6.**
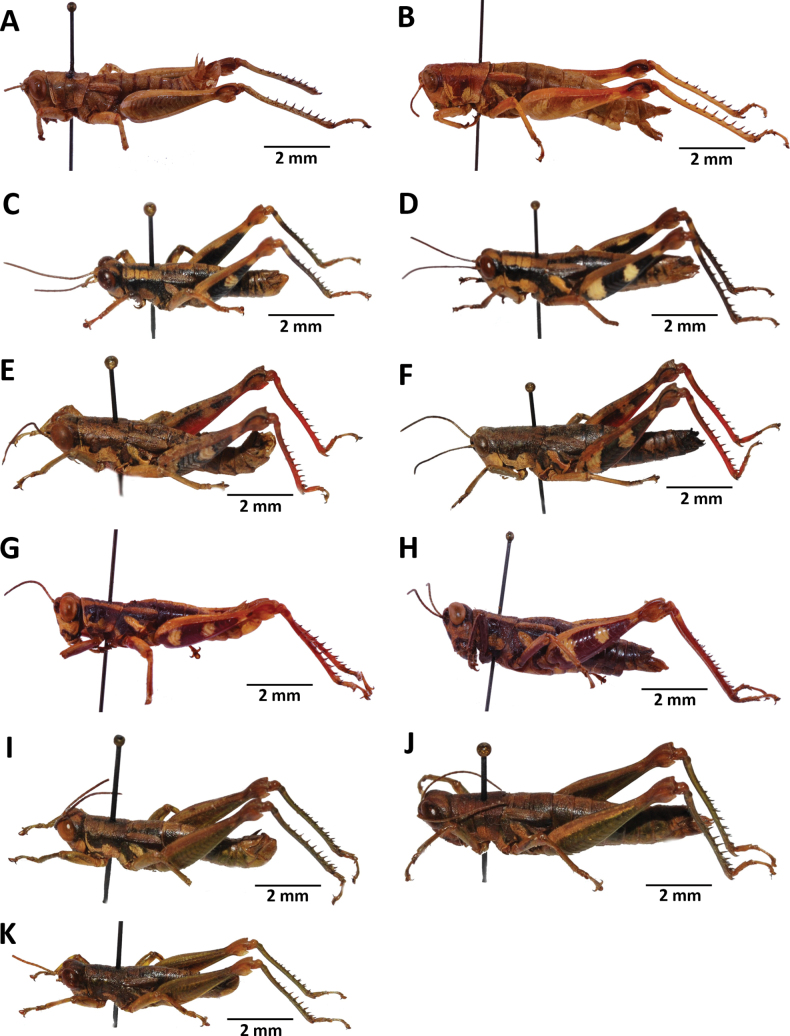
Images of holotypes, allotypes, letotype and neallotype of *Pteropera* species in lateral view **A***P.spleniata* (holotype ♂) **B***P.spleniata* (holotype ♀) **C***P.teocchii* (holotype ♂) **D***P.teocchii* (allotype ♀) **E***P.thibaudi* (holotype ♂) **F***P.thibaudi* (allotype ♀) **G***P.verrucigena* (lectotype ♂) **H***P.verrucigena* (♀) **I***P.villiersi* (holotype ♂) **J***P.villiersi* (allotype ♀) **K***P.uniformis* (neallotype ♂).

**Figure 7.**
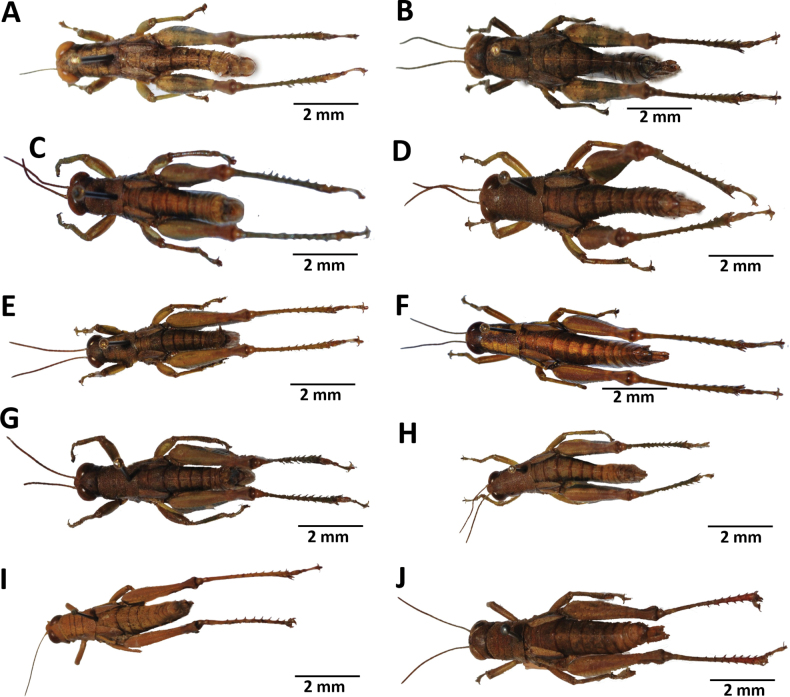
Images of holotypes, allotypes and paratype of *Pteropera* species in dorsal view **A***P.augustini* (holotype ♂) **B***P.augustini* (allotype ♀) **C***P.balachowskyi* (holotype ♂) **D***P.balachowskyi* (allotype ♀) **E***P.bertii* (holotype ♂) **F***P.bertii* (paratype ♀) **G***P.brosseti* (holotype ♂) **H***P.brosseti* (allotype ♀) **I***P.basilewskyi* (paratype ♀) **J***P.bredoi* (holotype ♀).

**Figure 8.**
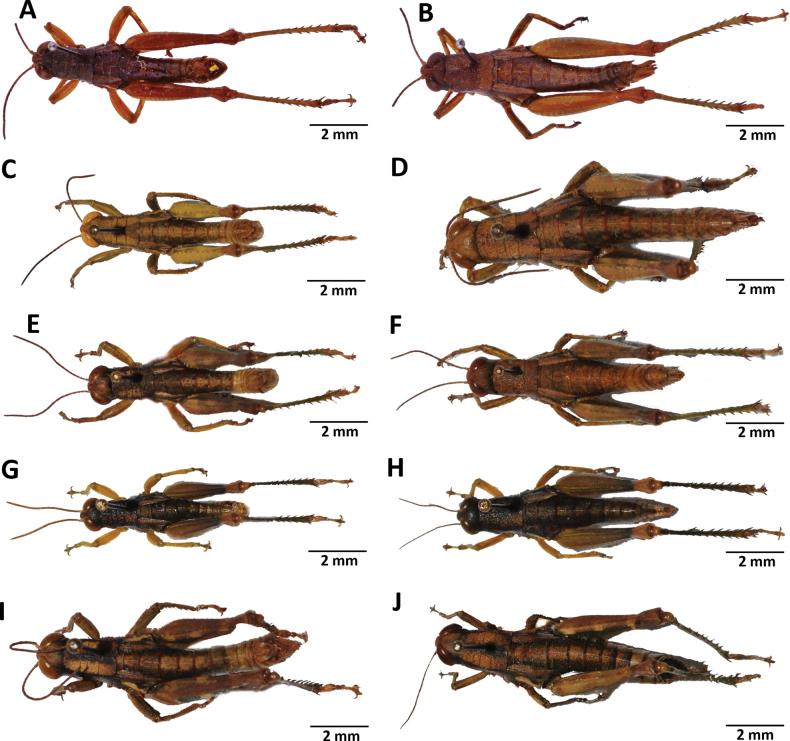
Images of holotypes, allotypes and paratype of *Pteropera* species in dorsal view **A***P.carnapi* (holotype ♂) **B***P.carnapi* (paratype ♀) **C***P.congoensis* (holotype ♂) **D***P.congoensis* (allotype ♀) **E***P.cornici* (holotype ♂) **F***P.cornici* (allotype ♀) **G***P.descampsi* (holotype ♂) **H***P.descampsi* (allotype ♀) **I***P.descarpentriesi* (holotype ♂) **J***P.descarpentriesi* (allotype ♀).

**Figure 9.**
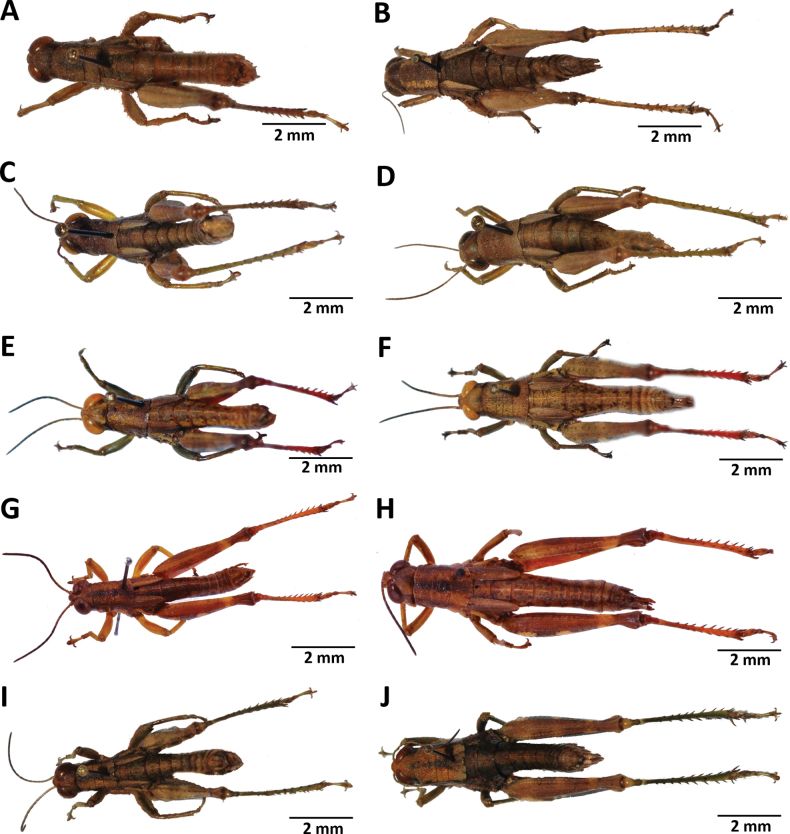
Images of holotypes, allotypes and paratype of *Pteropera* species in dorsal view **A***P.grilloti* (holotype ♂) **B***P.meridionalis* (holotype ♀) **C***P.jeanninae* (holotype ♂) **D***P.jeanninae* (allotype ♀) **E***P.karschikarschi* (paratype ♂) **F***P.karschikarschi* (paratype ♀) **G***P.karschizenkeri* (holotype ♂) **H***P.karschizenkeri* (allotype ♀) **I***P.menieri* (holotype ♂) **J***P.menieri* (allotype ♀).

**Figure 10.**
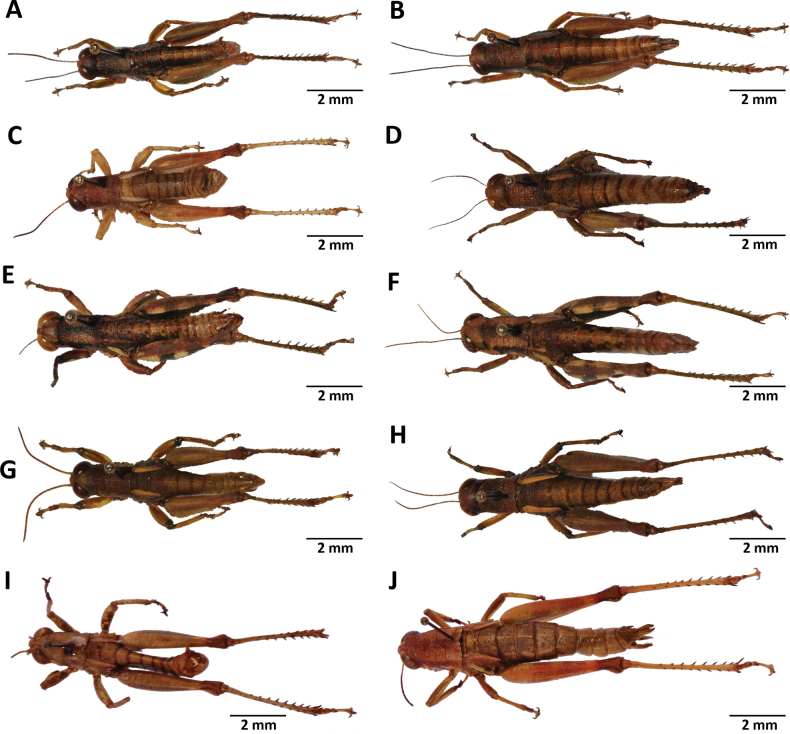
Images of holotypes, allotypes and paratype of *Pteropera* species in dorsal view **A***P.mirei* (holotype ♂) **B***P.mirei* (allotype ♀) **C***P.morini* (holotype ♂) **D***P.morini* (allotype ♀) **E***P.pillaulti* (holotype ♂) **F***P.pillaulti* (allotype ♀) **G***P.poirieri* (holotype ♂) **H***P.poirieri* (allotype ♀) **I***P.spleniata* (holotype ♂) **J***P.spleniata* (holotype ♀).

**Figure 11.**
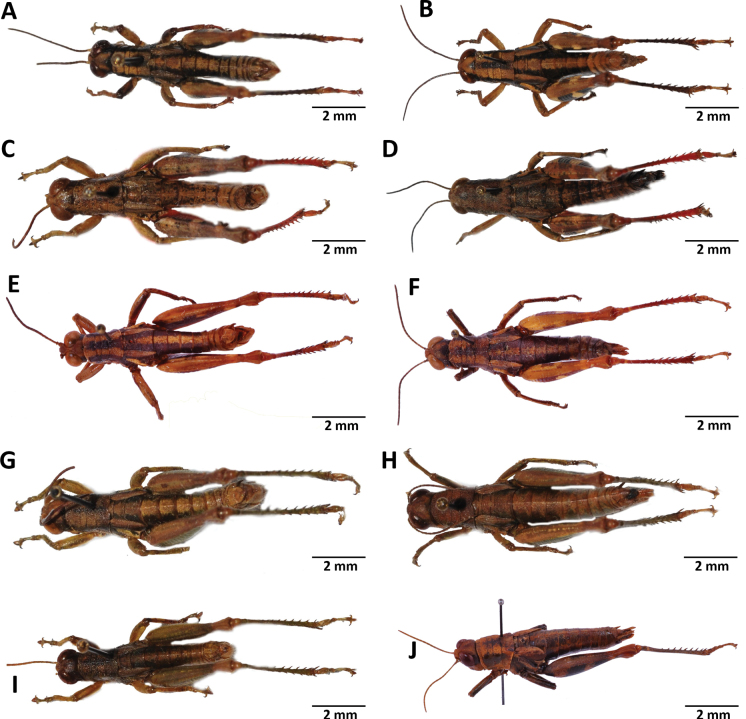
Images of holotypes, allotypes, neallotype and paratype of *Pteropera* species in dorsal view **A***P.teocchii* (holotype ♂) **B***P.teocchii* (allotype ♀) **C***P.thibaudi* (holotype ♂) **D***P.thibaudi* (allotype ♀) **E***P.verrucigena* (holotype ♂) **F***P.verrucigena* (allotype ♀) **G***P.villiersi* (holotype ♂) **H***P.villiersi* (allotype ♀) **I***P.uniformis* (neallotype ♂) **J***P.femorata* (♀).

The complete list of the currently known *Pteropera* species and subspecies with the specimen ID, type category, collection location, date of collection and depository, are presented in Table 2. In addition, we present the images of the holotypes, allotypes, paratypes, and neallotypes of each species whenever possible (Figs 2–11). In the case of *P.femorata*, we downloaded existing photographs of the type specimen from the Orthoptera Species File website (Cigliano et al. 2024).

**Table 2.** Complete list of currently known *Pteropera* species and subspecies considered in the present study.

N°	ID	Species	Type	Sex	Location	Author, year	Coll. date	Depository
**1**	MNHN-EO-CAELIF11462	*Pteroperaaugustini*	Holotype	m	Near Youmbi, Gabon	Donskoff, 1981	11.06.1974	MNHN
	MNHN-EO-CAELIF11463	*Pteroperaaugustini*	Allotype	f	Near Youmbi, Gabon	Donskoff, 1981	11.06.1974	MNHN
**2**	MNHN-EO-CAELIF11013	*Pteroperabalachowskyi*	Holotype	m	Between Mimomgo and Koulamouto, Gabon	Donskoff, 1981	15.06.1974	MNHN
	MNHN-EO-CAELIF11014	*Pteroperabalachowskyi*	Allotype	f	Between Mimomgo and Koulamouto, Gabon	Donskoff, 1981	15.06.1974	MNHN
**3**	MNHN-EO-CAELIF11461	*Pteroperabasilewskyi*	Paratype	f	Sankuru: Komi, Congo Museum	Donskoff, 1981	12.1913	RMCA
**4**	MNHN-EO-CAELIF11246	*Pteroperabertii*	Holotype	m	Nkoemvone, Cameroon	Donskoff, 1981	10–14.11.1975	MNHN
	MNHN-EO-CAELIF11247	*Pteroperabertii*	Allotype	f	Nkoemvone, Cameroon	Donskoff, 1981	10–14.11.1975	MNHN
**5**	NA	*Pteroperabredoi*	Holotype	f	Kalulu	Donskoff, 1981	06.05.1939	RBINS
**6**	MNHN-EO-CAELIF11197	*Pteroperabrosseti*	Holotype	m	Ipassa Quadrat, Gabon	Donskoff, 1981	3–30.05.1974	MNHN
	MNHN-EO-CAELIF11198	*Pteroperabrosseti*	Allotype	f	Ipassa Quadrat, Gabon	Donskoff, 1981	3–30.05.1974	MNHN
**7**	BA000180S01 DORSA	*Pteroperacarnapi*	Holotype	m	Yaoundé, Cameroon	Ramme, 1929	11.97	MfN
	BA000180S02 DORSA	*Pteroperacarnapi*	Paratype	f	Yaoundé, Cameroon	Ramme, 1929	11.97	MfN
**8**	MNHN-EO-CAELIF11162	*Pteroperacongoensis*	Holotype	m	Dimonika, Congo	Donskoff, 1981	01.1964	MNHN
	MNHN-EO-CAELIF11163	*Pteroperacongoensis*	Allotype	f	Dimonika, Congo	Donskoff, 1981	01.1964	MNHN
**9**	MNHN-EO-CAELIF11249	*Pteroperacornici*	Holotype	m	N´Go, Congo-Brazzaville	Donskoff, 1981	12.03.1973	MNHN
	MNHN-EO-CAELIF11253	*Pteroperacornici*	Allotype	f	N´Go, Congo-Brazzaville	Donskoff, 1981	12.03.1973	MNHN
**10**	MNHN-EO-CAELIF11456	*Pteroperadescampsi*	Holotype	m	Ongot (Yaoundé), Cameroon	Donskoff, 1981	2–4.11.1975	MNHN
	MNHN-EO-CAELIF11457	*Pteroperadescampsi*	Allotype	f	Ongot (Yaoundé), Cameroon	Donskoff, 1981	2–4.11.1975	MNHN
**11**	MNHN-EO-CAELIF11031	*Pteroperadescarpentriesi*	Holotype	m	Odzala, Congo	Donskoff, 1981	10.1963	MNHN
	MNHN-EO-CAELIF11032	*Pteroperadescarpentriesi*	Allotype	f	Odzala, Congo	Donskoff, 1981	10.1963	MNHN
**12**	NA	*Pteroperafemorata*	Holotype	f	Boko, Congo-Brazzaville	(Giglio-Tos, 1907)	-	EMT
**13**	MNHN-EO-CAELIF11493	*Pteroperagrilloti*	Holotype	m	N´Gongo, Congo-Brazzaville	Donskoff, 1981	25.02.1970	MNHN
**14**	MNHN-EO-CAELIF10990	*Pteroperajeanninae*	Holotype	m	Between Okondja and Odzala, Gabon	Donskoff, 1981	18.06.1974	MNHN
	MNHN-EO-CAELIF10991	*Pteroperajeanninae*	Allotype	f	Between Okondja and Odzala, Gabon	Donskoff, 1981	18.06.1974	MNHN
**15**	MNHN-EO-CAELIF11442	*Pteroperakarschikarschi*	NA	m	Mvoum, Gabon	(I. Bolívar, 1905)	1–15.11.1969	MNHN
	MNHN-EO-CAELIF11443	*Pteroperakarschikarschi*	NA	f	Mont cristal, Gabon	(I. Bolívar, 1905)	3.06.1974	MNHN
**16**	BA000178S01 DORSA	*Pteroperakarschizenkeri*	Holotype	m	Bipindi, Cameroon	Ramme, 1929	02.1904	MfN
	BA000178S06 DORSA	*Pteroperakarschizenkeri*	Allotype	f	Bipindi, Cameroon	Ramme, 1929	11.04.1897	MfN
**17**	MNHN-EO-CAELIF11531	*Pteroperamenieri*	Holotype	m	Dimonika, Congo-Brazzaville	Donskoff, 1981	18.02.1978	MNHN
	MNHN-EO-CAELIF11532	*Pteroperamenieri*	Allotype	f	Dimonika, Congo-Brazzaville	Donskoff, 1981	18.02.1978	MNHN
**18**	NA	*Pteroperameridionalis*	Holotype	f	Kwango (Popokabak), Coll-Mus-Congo	Donskoff, 1981	1949	Tervuren Museum
**19**	MNHN-EO-CAELIF11241	*Pteroperamirei*	Holotype	m	Nkoemvone	Donskoff, 1981	10–14.11.1975	MNHN
	MNHN-EO-CAELIF11242	*Pteroperamirei*	Allotype	f	Nkoemvone	Donskoff, 1981	10–14.11.1975	MNHN
**20**	MNHN-EO-CAELIF11238	*Pteroperamorini*	Holotype	m	M´Be, Congo-Brazzaville	Donskoff, 1981	03.1973	MNHN
	MNHN-EO-CAELIF11239	*Pteroperamorini*	Allotype	f	M´Be, Congo-Brazzaville	Donskoff, 1981	5.01.77	MNHN
**21**	MNHN-EO-CAELIF11232	*Pteroperapillaulti*	Holotype	m	Djoumouna (Yaka-Yaka), Congo	Donskoff, 1981	4.01.1977	MNHN
	MNHN-EO-CAELIF11233	*Pteroperapillaulti*	Allotype	f	Djoumouna (Yaka-Yaka), Congo	Donskoff, 1981	4.01.1977	MNHN
**22**	MNHN-EO-CAELIF11501	*Pteroperapoirieri*	Holotype	m	M´Bomo, Congo	Donskoff, 1981	8.02.1977	MNHN
	MNHN-EO-CAELIF11502	*Pteroperapoirieri*	Allotype	F	M´Bomo, Congo	Donskoff, 1981	8.02.1977	MNHN
**23**	BA000177S01 DORSA	*Pteroperaspleniata*	Holotype	m	Chinchoxo, Congo	(Karsch, 1896)	NA	MfN
	BA000177S02 DORSA	*Pteroperaspleniata*	Holotype	f	Chinchoxo, Congo	(Karsch, 1896)	NA	MfN
**24**	MNHN-EO-CAELIF11444	*Pteroperateocchii*	Holotype	m	La Maboke, RCA	Donskoff, 1981	16.01.1968	MNHN
	MNHN-EO-CAELIF11245	*Pteroperateocchii*	Allotype	f	La Maboke, RCA	Donskoff, 1981	16.01.1968	MNHN
**25**	MNHN-EO-CAELIF11509	*Pteroperathibaudi*	Holotype	m	Vouka (Mossendjo), Congo-Brazzaville	Donskoff, 1981	02.1974	MNHN
	MNHN-EO-CAELIF11510	*Pteroperathibaudi*	Allotype	f	Vouka (Mossendjo), Congo-Brazzaville	Donskoff, 1981	02.1974	MNHN
**26**	MNHN-EO-CAELIF11248	*PteroperaUniformis*	Neallotype	m	Nkoemvone, Cameroon	Bruner, 1920	10–14.11.1975	MNHN
**27**	BA000175S01 DORSA	*Pteroperaverrucigena*	Lectotype	H	Barombi Station, Cameroon	Karsch, 1891	NA	MfN
	BA000175S02 DORSA	*Pteroperaverrucigena*	Allotype	f	Barombi Station, Cameroon	Karsch, 1891	NA	MfN
**28**	MNHN-EO-CAELIF11243	*Pteroperavilliersi*	Holotype	m	Sibiti, Congo	Donskoff, 1981	11.1963	MNHN
	MNHN-EO-CAELIF11244	*Pteroperavilliersi*	Allotype	f	Sibiti, Congo	Donskoff, 1981	11.1963	MNHN
**29**	SMNK-ORTH-0000001	*Pteroperakennei* sp. nov.	Holotype	m	Somalomo (Dja), Cameroon	Yetchom & Husemann, 2024	10.04.2022	SMNK
**30**	SMNK-ORTH-0000002	*Pteroperamatzkei* sp. nov.	Holotype	m	Somalomo (Dja), Cameroon	Yetchom & Husemann, 2024	10.04.2022	SMNK
**31**	SMNK-ORTH-0000003	*Pteroperamissoupi* sp. nov.	Holotype	m	Iboti (Ebo forest), Cameroon	Yetchom & Husemann, 2024	07.01.2022	SMNK

ID: specimen ID; m: male; f: female; Coll. date: collection date; NA: not applicable.

**Table 3.** Taxon sampling and GenBank accession numbers for the sequenced markers.

Species	Voucher Codes	Coll. date	Country of origin	GenBank accession number			GenSeq nomenclature
				COI	16S	12S	
*P.augustini*	CM1205	11.04.2022	Cameroon	PP700650	PP708801	PP737690	Genseq-3 COI, 16S, 12S
*P.carnapi*	CM1361	12.06.2022	Cameroon	PP707812	PP708833	NA	Genseq-3 Genseq-3 COI, 16S
*P.carnapi*	CM1363	12.06.2022	Cameroon	PP707813	PP708802	PP737691	Genseq-3 Genseq-3 COI, 16S, 12S
*P.carnapi*	CM1364	12.06.2022	Cameroon	PP700651	PP708803	PP737728	Genseq-3 Genseq-3 COI, 16S, 12S
*P.carnapi*	CM1365	12.06.2022	Cameroon	PP700652	PP708804	PP737692	Genseq-3 COI, 16S, 12S
*P.carnapi*	CM1366	12.06.2022	Cameroon	PP700653	PP708805	PP737729	Genseq-3 COI, 16S, 12S
*P.carnapi*	CM1371	12.06.2022	Cameroon	NA	PP708806	PP737693	Genseq-3 16S, 12S
*P.carnapi*	CM1373	12.06.2022	Cameroon	PP700654	PP708834	PP737694	Genseq-3 COI, 16S, 12S
*P.descampsi*	CM140	20.03.2022	Cameroon	PP707814	PP708835	PP737695	Genseq-3 COI, 16S, 12S
*P.descampsi*	CM141	20.03.2022	Cameroon	PP700655	PP708807	PP737696	Genseq-3 COI, 16S, 12S
*P.descampsi*	CM142	20.03.2022	Cameroon	PP700656	PP708808	PP737730	Genseq-3 COI, 16S, 12S
*P.descampsi*	CM143	20.03.2022	Cameroon	PP707815	PP708809	PP737697	Genseq-3 COI, 16S, 12S
*P.descampsi*	CM144	20.03.2022	Cameroon	PP707816	PP708836	PP737698	Genseq-3 COI, 16S, 12S
*P.karschizenkeri*	CM1425	09.09.2018	Cameroon	NA	NA	PP737731	Genseq-3 12S
*P.kennei* sp. nov.	CM1131	10.04.2022	Cameroon	PP707817	PP708810	NA	Genseq-2 COI, 16S
*P.kennei* sp. nov.	CM1135	10.04.2022	Cameroon	PP707818	NA	PP737699	Genseq-2 COI, 12S
*P.kennei* sp. nov.	CM1136	10.04.2022	Cameroon	PP700657	NA	PP737700	Genseq-2 COI, 12S
*P.kennei* sp. nov.	CM1138	10.04.2022	Cameroon	PP707819	PP708811	NA	Genseq-2 COI, 16S
*P.kennei* sp. nov.	CM1183	11.04.2022	Cameroon	NA	PP708815	NA	Genseq-2 16S
*P.kennei* sp. nov.	CM1139	10.04.2022	Cameroon	NA	NA	PP737732	Genseq-2 12S
*P.kennei* sp. nov.	CM1141	10.04.2022	Cameroon	PP700658	PP708812	PP737733	Genseq-2 COI, 16S, 12S
*P.kennei* sp. nov.	CM1142	10.04.2022	Cameroon	PP700659	NA	NA	Genseq-2 COI
*P.kennei* sp. nov.	CM1143	10.04.2022	Cameroon	NA	PP708813	PP737734	Genseq-2 16S, 12S
*P.kennei* sp. nov.	CM1182	11.04.2022	Cameroon	PP700660	PP708814	NA	Genseq-2 COI, 16S
*P.matzkei* sp. nov.	CM1127	10.04.2022	Cameroon	PP700661	PP708816	PP737735	Genseq-2 COI, 16S, 12S
*P.matzkei* sp. nov.	CM1357	28.06.2022	Cameroon	PP700662	PP708817	NA	Genseq-2 COI, 16S
*P.matzkei* sp. nov.	CM1358	28.06.2022	Cameroon	PP700663	PP708818	PP737736	Genseq-2 COI, 16S, 12S
*P.matzkei* sp. nov.	CM1359	28.06.2022	Cameroon	PP700664	PP708819	PP737737	Genseq-2 COI, 16S, 12S
*P.missoupi* sp. nov.	CM139	20.03.2022	Cameroon	PP700665	PP708837	PP737738	Genseq-2 COI, 16S, 12S
*P.missoupi* sp. nov.	CM278	15.06.2020	Cameroon	PP700666	PP708820	PP737701	Genseq-2 COI, 16S, 12S
*P.missoupi* sp. nov.	CM280	15.06.2020	Cameroon	PP700667	PP708838	PP737702	Genseq-2 COI, 16S, 12S
*P.missoupi* sp. nov.	CM281	15.06.2020	Cameroon	PP700668	PP708821	PP737703	Genseq-2 COI, 16S, 12S
*P.missoupi* sp. nov.	CM367	05.12.2021	Cameroon	PP707820	PP708839	PP737739	Genseq-2 COI, 16S, 12S
*P.missoupi* sp. nov.	CM569	07.01.2022	Cameroon	PP700669	PP708822	PP737740	Genseq-2 COI, 16S, 12S
*P.missoupi* sp. nov.	CM570	07.01.2022	Cameroon	PP700670	PP708823	PP737741	Genseq-2 COI, 16S, 12S
*P.missoupi* sp. nov.	CM571	07.01.2022	Cameroon	PP700671	PP708824	PP737742	Genseq-2 COI, 16S, 12S
*P.missoupi* sp. nov.	CM1094	15.06.2020	Cameroon	PP700672	PP708825	PP737704	Genseq-2 COI, 16S, 12S
*P.uniformis*	CM373	05.12.2021	Cameroon	PP707821	PP708840	PP737705	Genseq-3 COI, 16S, 12S
*P.uniformis*	CM1187	11.04.2022	Cameroon	PP700673	NA	PP737743	Genseq-3 COI, 12S
*P.verrucigena*	CM1423	03.04.2017	Cameroon	PP707822	PP708826	PP737706	Genseq-3 COI, 16S, 12S
*P.verrucigena*	CM1424	04.04.2017	Cameroon	PP707823	PP708841	PP737707	Genseq-3 COI, 16S, 12S
*C.stramineus*	CM735	06.05.2020	Cameroon	PP700674	NA	NA	Genseq-3 COI
*C.stramineus*	CM973	09.04.2020	Cameroon	PP707824	NA	PP737744	Genseq-3 COI, 12S
*E.modica*	CM 342	05.12.2021	Cameroon	PP700675	PP708827	NA	Genseq-3 COI, 16S
*E.modica*	CM343	05.12.2021	Cameroon	PP700676	PP708842	NA	Genseq-3 COI, 16S
*E.modica*	CM344	05.12.2021	Cameroon	PP700677	PP708828	PP737745	Genseq-3 COI, 16S, 12S
*E.modica*	CM345	05.12.2021	Cameroon	PP700678	PP708843	NA	Genseq-3 COI, 16S
*P.notatus*	CM358	05.12.2021	Cameroon	PP700679	PP708829	NA	Genseq-3 COI, 16S
*P.notatus*	CM359	05.12.2021	Cameroon	PP707825	PP708830	PP737746	Genseq-3 COI, 16S, 12S
*P.notatus*	CM360	05.12.2021	Cameroon	PP700680	PP708831	NA	Genseq-3 COI, 16S
*P.notatus*	CM596	02.03.2021	Cameroon	PP700681	PP708832	NA	Genseq-3 COI, 16S
*P.notatus*	CM781	17.07.2018	Cameroon	PP700682	NA	NA	Genseq-3 COI

**Coll. date**: collection date.

#### ﻿ Pteropera augustini

Taxon classificationAnimaliaOrthopteraAcrididae

Donskoff, 1981

D1FDE9B2-026A-5D05-91A1-A486BFB75682

Figs 2A, B , 7A, B

Pteropera augustini Donskoff, 1981: 51–52.

##### Type materials examined.

***Holotype*** • ♂; Gabon. Near Youmi, in forest habitat; 0°24.617'N, 9°26.200'E; 11 Jun. 1974; M. Donskoff & J. Le Breton leg.; MNHN, MNHN-EO-CAELIF11462.

##### Other material examined.

Cameroon • 1 ♀, subadult; Somalomo, in the Dja Biosphere Reserve, cocoa farm; 3°23.650'N, 12°53.583'E, 606 m a.s.l.; 11 Apr. 2022; J.A. Yetchom Fondjo leg.; SMNK.

##### Morphological characteristics.

Two subocellar facial spots; posterior margin of the pronotum slightly indented; no marked difference between the upper half and lower half of the elytra; shiny black line along the lower margin; metathoracic episternites almost entirely pale; outer area of the hind femora green with pale spots, widely spread on the upper carina, median spot rounded, small, basal spot triangular; lower basal half of the inner area greenish-brown, pale spot rounded and small; only the lower inner area brownish; hind tibiae green.

Female subgenital plate pentagonal; egg-guide short; anterior apodemes narrow, short; medio-dorsal pocket narrow; basivalvar sclerites broad, slightly fused, almost perpendicular to each other; end of the copulatory bursa enlarged in the shape of a bubble; spermatheca ampulla arched, both diverticula of different diameters; recurrent distal trunk of the lateral diverticulum 4–5× longer than the proximal trunk.

##### Remarks.

This species is known by both males and females. The last female subadult stage was collected during this study and represents the first signalization of this species in Cameroon.

##### Distribution.

Gabon; Cameroon (Fig. 16A).

#### ﻿ Pteropera carnapi

Taxon classificationAnimaliaOrthopteraAcrididae

Ramme, 1929

5D235732-67F5-5BF1-8ED3-BEC76DDB2983

Figs 3A, B , 8A, B

Pteropera biloloca Sjöstedt 1931: 28; Dirsh 1965: 339.
Pteropera carnapi Ramme 1929: 364; Donskoff 1981: 59.

##### Type material examined.

***Holotype*.** Cameroon • ♂; Yaoundé; 3°50.883'N, 11°30'7"E; Jun. 1887; V. Carnap leg.; MfN, BA000180S01-DORSA.

##### Other material examined.

Cameroon • 4 ♀♀; Ongot; 3°42.517'N, 11°15.167'E; 15 Jun. 2020; J.A. Yetchom Fondjo leg.; SMNK. Cameroon • 2 ♂♂; Iboti, Ebo forest; 4°27.001'N, 10°27.002'E, 731 m a.s.l.; 7 Jan. 2022; J.A. Yetchom Fondjo leg.; SMNK. Cameroon • 9 ♂♂, 4 ♀♀; Deng-Deng National Park; 3°21.364'N, 12°44.615'E, 731 m a.s.l.; 12 Jun. 2022; A.R. Nzoko Fiemapong leg.; SMNK.

##### Redescription.

Frontal ridge raised above the median ocelli, prominent between the antennae; head and pronotum with well contrasted pale and dark colors; subocellar facial spot large; lower margin of elytra shiny black; meso- and metathoracic episternites yellow in their center; front legs, ventral area of hind femora yellow-green; dorsal area of the body and upper areas of hind femora more or less dark brown; hind tibiae bluish-green; male pallium and supra-anal plate raised; male cerci bilobate, with inner lobe equal to or longer than the outer lobe. ***Epiphallus*** (Fig. 15A): Ancorae small and closer to each other; bridge arched; anterior projections prominent, triangular. ***Phallic complex*** (Fig. 15B–D): dorsal arch of cingulum U-shaped, apodemes slender, clearly overhanging or exceeding level of separation of endophallic valves, with strongly incurved apex; upper ectophallic sheath very long in profile; lower ectophallic sheath not covering or enveloping the base of the rami; rami not bent; aedeagus valves in the form of a thin blade; latero-ventral sclerite triangular.

##### Distribution.

Cameroon; Central African Republic; Gabon; Congo (Fig. 16B).

#### ﻿ Pteropera descampsi

Taxon classificationAnimaliaOrthopteraAcrididae

Donskoff, 1981

B4E61878-86E4-5B7E-B792-9315180E6D04

Figs 3G, H , 8G, H

##### Type materials examined.

***Holotype*.** Cameroon • ♂; Yaoundé, Ongot [Onguot]; 3° 50.899'N, 11°30.133'E; 2–4 Nov. 1975; M. Descamps leg.; MNHN, MNHN-EO-CAELIF11456.

##### Other material examined.

Cameroon • 4 ♂♂, 3 ♀♀; Ongot; 3°51.517'N, 11°22.367'E; 15 Jun. 2020; J.A. Yetchom Fondjo & A.R. Nzoko Fiemapong leg.; SMNK. Cameroon • 1 ♀; Meyomessala; 3°6.431'N, 12°14.703'E; 18 Aug. 2021; J.A. Yetchom Fondjo leg.; SMNK). Cameroon • 5 ♀♀; Ongot; 3°51.517'N, 11°22.367'E; 5 Dec. 2021; J.A. Yetchom Fondjo leg.; SMNK. Cameroon • 6 ♂♂, 2 ♀♀; Ongot; 3°51.517'N, 11°22.367'E; 20 Mar. 2022; J.A. Yetchom Fondjo & A.R. Nzoko Fiemapong leg.; SMNK.

##### Redescription.

Subocellar facial spot V-shaped, wide; dark median longitudinal band on the pronotum disc not very distinct; the two contiguous pale bands present; lower part of body very pale on living specimen; pronotum disc shiny brownish; pale basal posterior spots on the lateral lobes of the pronotum extending almost to the lower margin; lower half of elytra shiny black, upper half brown; meso- and metathoracic episternites with pale, narrow, median and basal band; front and middle legs pale green; outer and inner sides of hind femora pale, greenish yellow with apical third gradually darkening towards the pregenicular black ring; knees pale brown; hind tibiae green, sometimes very dark; male subgenital plate truncated; male cerci with a small inner preapical lobe. ***Epiphallus*** (15E): of smaller size, ancorae small, anterior projections and lateral plates wide. ***Phallic complex*** (Fig. 15F–H): dorsal arch of cingulum V-shaped, open, apodemes reaching apex of endophallic sclerites; rami bent; upper ectophallic sheath short; lower ectophallic sheath not capping base of rami; latero-ventral sclerite subtriangular.

##### Distribution.

Cameroon (Fig. 16B).

#### ﻿ Pteropera karschi zenkeri

Taxon classificationAnimaliaOrthopteraAcrididae

Ramme, 1929

689C1039-EF94-52E3-A987-D1FF6F883AA4

Figs 4I, J , 9G, H

##### Type material examined.

***Holotype*.** Cameroon • ♂; Bipindi, “Urwald”; 3°4.657'N, 10°24.607'E; Sep. 1898; G. Zenker leg.; MfN, BA000178S01-DORSA.

##### Other material examined.

Cameroon • 2 ♂♂, 1 ♀; Koukoué, on shrubs in palm plantations; 4°2.400'N, 10°7.002'E; 19 Sep. 2018; J.A. Yetchom Fondjo leg.; SMNK.

##### Redescription.

**Male**: medium size; generally greenish; tegument weakly granular; head conical and oblique; fastigium of vertex short with obtuse apex; interocular space narrow; pronotum rugous, without lateral carinae, with straight median carina crossed by three furrows and with rounded posterior margin slightly indented in the middle, anterior margin incised in the middle; longitudinal median band of the pronotal disc darker and wider than the adjoining clear bands; prozona longer than the metazona; prosternal tubercle short conical and flattened at the base, forming an outline of a collar with the prothoracic presternite; mesosternal space open and longer than its wide; dorsal carina of hind femur finely toothed, distal end pointed; arolium large; outer area of hind femora with three pale spots; inner areas of hind femora with a median pale spot; inner and lower areas of hind femora, hind tibiae orange; middle area of male supra-anal plate with a transverse groove and tubercles on the sides; basal area with two digital tubercles; male subgenital plate conical, with a short tubercle at the apex; male cerci long conical, extending beyond the end of the supra-anal plate, with a wide internal pre-apical lobule. ***Epiphallus*** (Fig. 15I): bridge short and arched, convex; ancorae well developed, curved inwards and with an obtuse apex; anterior projections narrow, triangular; lophi broadly lobiform, slightly curved anteriorly; lateral plates broad, subparallel; oval sclerite large. ***Phallic complex*** (Fig. 15J–L): dorsal arch of cingulum U-shaped, closed, not rectangular; rami of cingulum not angular; apodemes thin and very short, reaching only the end of ejaculatory sac with incurved apices; endophallic apodemes short; aedeagus curved upward, straight, oblique in lateral view; lower ectophallic sheath not enveloping the base of rami.

**Female**: subgenital plate pentagonal; egg-guide short; anterior apodemes short, narrow, with projecting posterior margin; valves of ovipositor robust, curved towards the apex; external margins of the dorsal valves saw-toothed; spermatheca with medium-sized axial diverticulum; distal trunk, recurrent of the lateral diverticulum of the spermatheca 1.5× longer than the proximal trunk.

##### Remarks.

Donskoff distinguished *Pteroperakarschikarschi* from *Pteroperakarschizenkeri* on the basis of external morphology and described the genitalia structures of *P.karschikarschi* as representative of both subspecies. *Pteroperakarschizenkeri* resembles *P.karschikarschi* in several genitalia features but can easily be distinguished by a convex epiphallus bridge (concave in *P.karschikarschi*), with the apex of the aedeagus curved upward, oblique in lateral view (horizontal, in line with valves in *P.karschikarschi*), apodemes of the cingulum curved inwards in its apical part (straight in *P.karschikarschi*); and the distal trunk of the lateral diverticulum of the spermatheca being 1.5× longer than the proximal trunk (5–6× longer than the proximal trunk in *P.karschikarschi*). The juvenile of this species is unknown.

##### Distribution.

Cameroon; Equatorial Guinea; Gabon (Fig. 17B).

#### ﻿ Pteropera uniformis

Taxon classificationAnimaliaOrthopteraAcrididae

Bruner, 1920

D9F61B9C-B2E3-504F-966C-1A8054E44122

Figs 6K , 11I , 15M–P

##### Type material examined.

***Holotype*.** Cameroon • ♂; Batanga; 2°50.795'N, 9°53.699'E; Apr. 1914; F.H. Hope leg.; ANSP.

##### Other material examined.

Cameroon • 1 ♂; Ongot; 3°51.517'N, 11°22.367'E; 5 Dec. 2021; J.A. Yetchom Fondjo leg.; SMNK. Cameroon • 1 ♂; Somalomo, in the Dja Biosphere reserve; 3°22.448'N, 12°43.990'E; 11 Apr. 2022; J.A. Yetchom Fondjo leg.; SMNK.

##### Redescription.

Lower side of the body clear; median dark band on pronotum disc narrow, the two contiguous clear bands faintly marked; posterior basal spot on the lateral lobes of pronotum narrow, not reaching the lower edge; metathoracic episternite with a straight, median stripe limited to the base of the segment; lower half of elytra shiny black, upper half brown; front and middle legs pale green. Inner and outer areas of posterior female hind femora pale, greenish yellow, with small pregenicular black ring; knees pale brown; posterior tibiae green, sometimes very dark; male cerci with small internal preapical lobule. ***Epiphallus*** (Fig. 15M): bridge thin, narrow; anterior projections lobiform. ***Phallic complex*** (Fig. 15N–P): dorsal arch of cingulum rounded, almost firm, at the level of the ejaculatory sac, leaving endophallic sclerites almost entirely free, very divergent anteriorly; rami not bent; upper ectophallic sheath long; latero-ventral sclerite in profile, elbowed.

##### Remarks.

The juveniles of this species is unknown.

##### Distribution.

Cameroon (Fig. 18B).

#### ﻿ Pteropera verrucigena

Taxon classificationAnimaliaOrthopteraAcrididae

Karsch, 1891

0A2940B8-308D-5473-89D9-645345E729B7

Figs 6G, H , 11E, F

##### Type material examined.

***Lectotype*.** Cameroon • ♂; Barombi Station; 4°40.016'N, 9°22.999'E; Dr. Paul Preuss leg.; MfN, BA000175S01-DORSA.

##### Other material examined.

Cameroon • 2 ♂♂, 1 ♀; Sohock; 4°57.250'N, 10°14.833'E; 3 Apr. 2017; J.A. Yetchom Fondjo leg.; SMNK.

##### Redescription.

**Male**: medium size, integument rugous; head conical and oblique; fastigium of vertex short with obtuse apex; eyes prominent and globose; antenna, filiform longer than head and pronotum combined; pronotum without lateral carinae and with straight median carina, crossed by three sulci, its anterior and posterior margins rounded and incised in the middle; pale basal band of lateral lobes of pronotum narrowed in front of second transverse furrow but not interrupted; longitudinal median band of pronotum disc dark and less wider than adjacent clear bands; prozona longer than metazona; prosternal tubercle conical; anterior margin of mesosternum broadly projected medially; mesosternal space open and longer than it is wide; elytra vestigial or lobiform; median pale spot on inner area of hind femora absent; outer area of hind femora with three pale spots; incipient spots along medio-superior margin at level of outer spots present; dorsal carina of hind femora finely toothed; lower outer areas of hind femora dark, wine-colored; hind tibiae wine-colored; distal half of hind tibiae widened, basal ring present; supra-anal plate subconical, with two digital tubercles near lateral margins; subgenital plate short conical, gradually tapering towards rounded apex; cerci conical, curved inward and without preapical lobule. ***Epiphallus*** (Fig. 15Q): bridge narrow, short and arched; ancorae small, close together and with acute apex; lophi short, broad and lobiform; lateral plates broad and rounded; anterior and posterior projections short. ***Phallic complex*** (Fig. 15R–T): dorsal arch of the cingulum V-shaped, strongly open not overlying the endophallic sclerites; latero-ventral sclerite broad, subtriangular; zygoma wide; apodemes of the cingulum long, reaching the apex of the endophallic apodemes; lower ectophallic sheath not enveloping the base of the rami; upper ectophallic sheath globular, sloping forward.

**Female**: Similar to the male but larger; supra-anal plate conical with a transverse groove in the middle field; posterior edge of the subgenital plate projecting; cercus conical with angular apex; dorsal valves of the ovipositor weakly toothed; base of the spermathecal duct widened well before it opened into the copulatory bursa; spermatheca ampulla relatively thin; distal, recurrent trunk of the spermatheca lateral diverticulum > 3× longer than the proximal trunk.

##### Remarks.

*Pteroperaverrucigena* was originally described from Barombi station (southwest Cameroon) with some paratypes recorded between Kumba-Mamfé (Southwest Cameroon) by Karsch (1891). We now additionally recorded the species from Sohock (Littoral Cameroon).

##### Distribution.

Cameroon (Fig. 18B).

#### ﻿ Pteropera kennei

Taxon classificationAnimaliaOrthopteraAcrididae

Yetchom & Husemann sp. nov.

649D9392-8656-5941-890E-7162AD71835F

https://zoobank.org/12D96BC6-86C8-4CA3-A0ED-F5887640A268

Fig. 12A–M

##### Type material examined.

***Holotype*.** Cameroon • ♂; Somalomo, in the forest along the Dja River; 3°22.448'N, 12°43.990'E, 606 m a.s.l.; 10 Apr. 2022; J.A. Yetchom Fondjo leg.; SMNK, SMNK-ORTH-0000001. ***Paratypes*.** Cameroon • 16 ♂♂, 3 ♀♀; Somalomo, in the forest along the Dja River; 3°22.448'N, 12°43.990'E, 606 m a.s.l.; 10–11 Apr. 2022; J.A. Yetchom Fondjo & A.R. Nzoko-Fiemapong leg.; SMNK, MNHN. Cameroon • 1 ♂; Deng-Deng National Park; 3°21.364'N, 12°44.615'E, 661 m a.s.l.; 12 Jun. 2022; A.R. Nzoko-Fiemapong leg.; SMNK.

##### Diagnosis.

*Pteroperakennei* sp. nov. is similar to *P.uniformis* Bruner, 1920, from Cameroon in terms of its general coloration, a dark longitudinal band and contiguous pale bands on the pronotum disc and the outer area of hind femora without pale spots. However, the new species can easily be distinguished from *P.uniformis* (Figs 6K, 11I) by its lateral lobes of pronotum without a pale basal band (present in *P.uniformis* as well as in all other *Pteropera* species); its more or less pale green coloration on the hind femora (inner and outer sides of hind femora greenish yellow in *P.uniformis*); male genitalia differ by its closed dorsal arch of cingulum (strongly open in *P.uniformis*); aedeagus horizontal apically, in line with valves (anteriorly sloping in *P.uniformis*); female genitalia differ by egg-guide being slender (broad in *P.uniformis*); and basivalvar sclerites forming an obtuse angle (acute angle in *P.uniformis*).

The new species is also similar to *Pteroperadescampsi* Donskoff, 1981, from which it can be distinguished by the following characteristics: a pale basal band on the lateral lobes of the pronotum is absent but present in *P.descampsi*; bilobed male cerci, whereas male cerci are short in *P.descampsi*; the pallium and subgenital plate in males are slightly raised, whereas the apex of the subgenital plate is truncated in *P.descampsi*; the apex of the aedeagus is horizontal, in line with valves, whereas the aedeagus is curved upwards, and the apex is divergent pallets in *P.descampsi*; the dorsal arc of the cingulum is closed, whereas it is strongly open in *P.descampsi*; and the basivalvar sclerites of the female subgenital plate are described as an obtuse angle (acute angle in *P.descampsi*).

##### Description.

**Figure 12.**
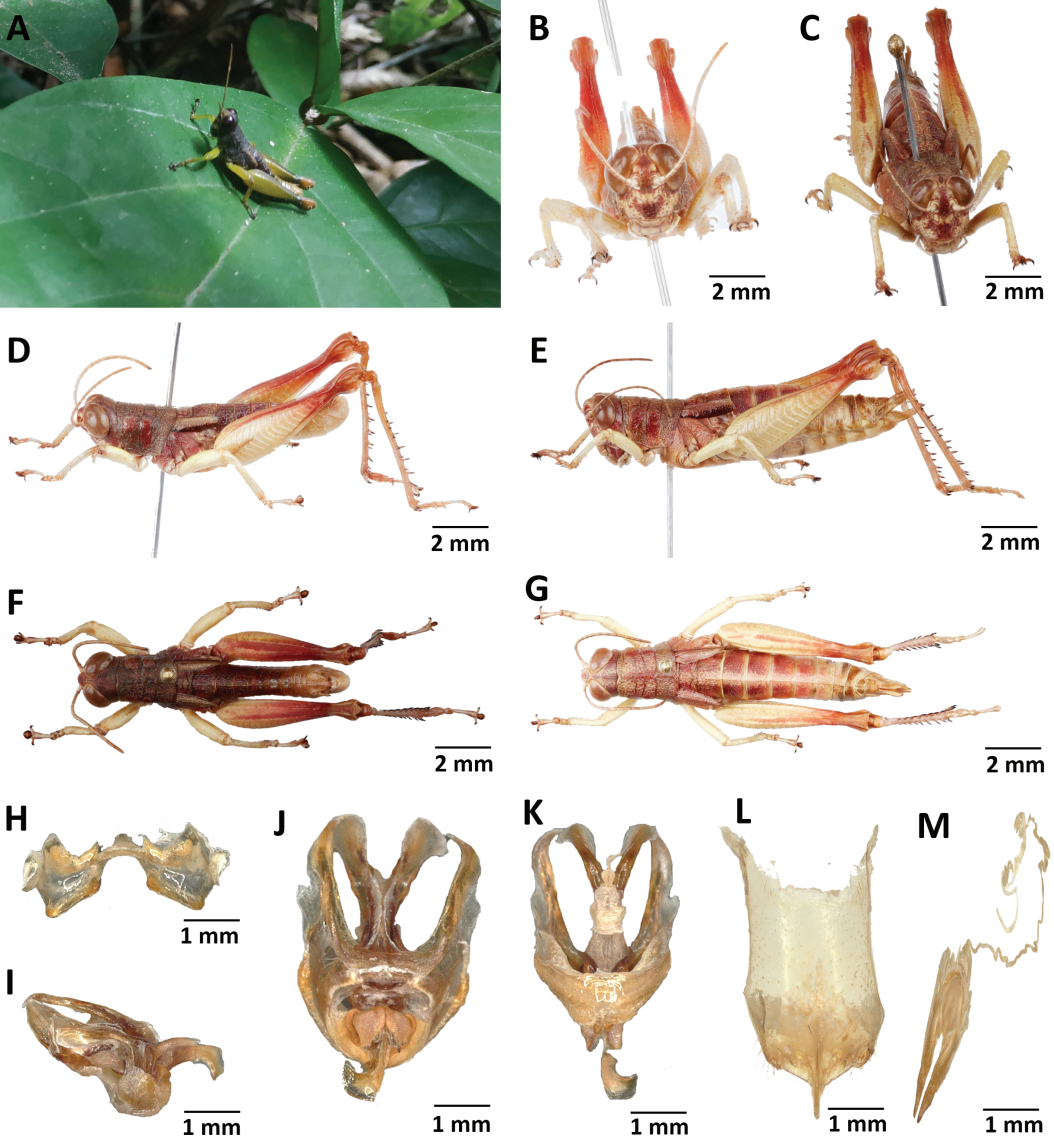
*Pteroperakennei* sp. nov. **A** habitus image of a male under natural conditions **B** male frontal view **C** female frontal view **D** male lateral view **E** female lateral view **F** male dorsal view **G** female dorsal view **H** epiphallus dorsal view **I** phallic complex lateral view **J** phallic complex dorsal view **K** phallic complex ventral view **L** female subgenital plate **M** female spermatheca.

**Male**: General coloration brown with pale green; body and legs with inconspicuous hairs, moderately rugous dorsally, smooth ventrally; eyes prominent; the large subocellar facial spot interrupted at the facial furrow, sometimes extending to the cheeks; antennae thin, filiform, longer than head and pronotum together; pronotum dark brown; dark longitudinal median band on pronotum disc present, wider than adjacent pale bands; basal pale bands on lateral lobes of pronotum absent; two incipient pale spots on the anterior margin of lateral lobes of pronotum; posterior margins of pronotum with or without incipient pale spots; median carina present and crossed dorsally by three sulci; lateral carinae absent; prozona longer than metazona; prosternal process short conical, compressed at its base; tegmina lobiform, only slightly reaching the third abdominal segment, lower half shiny black and upper half pale ochreous; mesosternal interspace open, ~ 1.3× longer than wide; meso- and metathoracic episternites dark brown; front and middle legs, inner and outer sides of hind femora pale green, with the apical third gradually darkening toward the knee; knee dark orange; dorsal basal lobes of hind femora longer than ventral ones; upper margins of hind femora with fine teeth; hind tibiae dark green, basal ring absent; external apical spines of hind tibiae absent; male cerci bilobed, the inner lobe being twice shorter than the outer; subgenital plate obtuse to rounded in dorsal view; pallium and supra-anal plate of male slightly raised. ***Epiphallus*** (Fig. 12H): small, bridge narrow, arched; lateral margins parallel; ancorae small, internally directed; lophi slender. ***Phallic complex*** (Fig. 12I–K): aedeagus small, short, curved; membranous apex of aedeagus, outside sheaths without sclerites, never filiform, and without ridge-like expansion; membranous apex of aedeagus outside sheaths, without sclerites, enlarged into a broad transverse lamina, never angular, never rolled up on itself; the dorsal arch of cingulum V-shaped, its apex acute, curved inwards; rami not bent, its lower part short; zygoma reduced; latero-ventral sclerites narrow; upper ectophallic sheath tight, short, slightly curved, with acute apex; upper eadeagus valve widened into a transverse blade; lower ectophallic sheath small, not enveloping the base of rami.

**Female**: As male, but larger; cerci short conical; valves of ovipositor narrow, > 3.5× longer than wide in coalescence position; subgenital plate (Fig. 12L) pentagonal, elongated, with truncated posterior margins; anterior apodemes narrow and short; egg-guide thin and long; ventral pockets of the vaginal floor large; copulatory bursa almost straight, gradually narrowing towards the front; bottom of the copulatory bursa close to the arc of the basivalvar sclerites; copulatory bursa above the basivalvar sclerites with a thick ventral gutter and membranous roof; each basivalvar sclerite barely curved, forming an obtuse angle; internal sclerite of the copulatory bursa short; the recurrent distal trunk of the lateral spermathecal diverticulum 3× longer than the proximal trunk; the base of the spermathecal duct opening at the apex of the bursa; spermathecal ampulla narrowed at the apex; spermathecal duct very long; axial diverticle of the spermatheca almost as long as the lateral diverticulum (Fig. 12M).

##### Measurements.

Males (mm) (*n* = 20): total length of body 11.81–19.83; length of pronotum 3.12–4.32; length of hind femur 11.23–12.53; length of elytra 3.24–4.27. Females (mm) (*n* = 5): total length of body 21.09–25.39; length of pronotum 4.59–5.37; length of hind femur 13.62–15.12; length of elytra 3.65–4.88; length of ovipositor 1.97–3.27. Detailed information is shown in Table 1.

##### Etymology.

The species was named in honor of Professor Martin Kenne in recognition of his work and scientific contribution to the biodiversity of insects in Cameroon.

##### Habitat.

Dense evergreen forest in the Congo Basin, Dja Biosphere Reserve, south Cameroon.

##### Distribution.

Cameroon, Somalomo in the Dja Biosphere Reserve and Deng-Deng National Park (Fig. 17B).

#### ﻿ Pteropera matzkei

Taxon classificationAnimaliaOrthopteraAcrididae

Yetchom & Husemann sp. nov.

A5A6BE9A-637E-5BE2-A8FB-D5DCF4BB8B5D

https://zoobank.org/B6C9D13F-A617-4895-82BB-794E2B5D0708

Fig. 13A–M

##### Type material examined.

***Holotype*.** Cameroon • ♂; Somalomo, in the forest along the Dja River; 3°22.448'N, 12°43.990'E, 602 m a.s.l.; 10 Apr. 2022; J.A. Yetchom Fondjo leg.; SMNK, SMNK-ORTH-0000002. ***Paratypes*.** Cameroon • 1 ♀; Somalomo, in the forest along the Dja River; 3°22.448'N, 12°43.990'E, 602 m a.s.l.; 10 Apr. 2022; J.A. Yetchom Fondjo leg.; SMNK. Cameroon • 3 ♂♂, 1 ♀; Somalomo, in the Dja Biosphere reserve; 3°22.448'N, 12°43.990'E, 602 m a.s.l.; 28 Jun. 2022; A.R. Nzoko-Fiemapong leg.; SMNK.

##### Diagnosis.

The new species *Pteroperamatzkei* sp. nov. is close to *Pteroperabertii* Donskoff, 1981 (Figs 2E, F, 7E, F) from Cameroon, from which it differs by the following characteristics: the entirely yellow coloration of meso- and metathoracic episternites (almost entirely pale in *P.bertii*); the dark brown coloration of hind femora and dark yellow coloration of front and middle legs, and hind tibiae (front and middle legs, hind femora, and hind tibiae are pale green in *P.bertii*); male genitalia differ in shape and size of phallic structures with the dorsal arch of the cingulum closed, long, extending beyond the apex of endophallic valves, and overhanging them apically (slightly open, not reaching apex of endophallic valves nor overhanging them in *P.bertii*); fore apodemes of the female subgenital plate thin, acute (broad, short in *P.bertii*); and the spermathecal ampulla elongate (broad apically in *P.bertii*).

*Pteroperamatzkei* sp. nov. differs from *Pteroperateocchii* Donskoff, 1981 (Figs 6C, D, 11A, B) in that the outer area of its hind femora has only one preapical spot (three spots, beginning of spots along the upper margin at the level of the outer spots in *P.teocchii*); dark yellow coloration of hind tibiae (black in *P.teocchii*); aedeagus slightly curved, describing less than a semicircle (strongly curved, describing an almost complete circle in *P.teocchii*); and female spermathecal ampulla elongated (enlarged at the junction of lateral and axial diverticula in *P.teocchii*).

The new species is also similar to *Pteroperaverrucigena* Karsch, 1891 (Figs 6G, H, 11E, F) from which it differs in the following characteristics: a single spot on the outer area of the hind femora, whereas *P.verrucigena* has three spots; dark yellow coloration of the fore and middle legs and hind tibiae, while the fore and middle legs in *P.verrucigena* are dark brown dorsally and wine red-colored ventrally, hind tibiae wine red-colored; membranous apex of aedeagus slightly curved, describing less than one semicircle, the tip short, whereas that of *P.verrucigena* is very curved, describing one semicircle with a long tip; and the dorsal arc of the cingulum is closed, long, extending beyond the apex of endophallic apodemes, whereas in *P.verrucigena*, the dorsal arc of the cingulum is open, not reaching the apex of endophallic apodemes; female spermathecal ampulla is elongated, whereas that of *P.verrucigena* is constricted apically.

##### Description.

**Figure 13.**
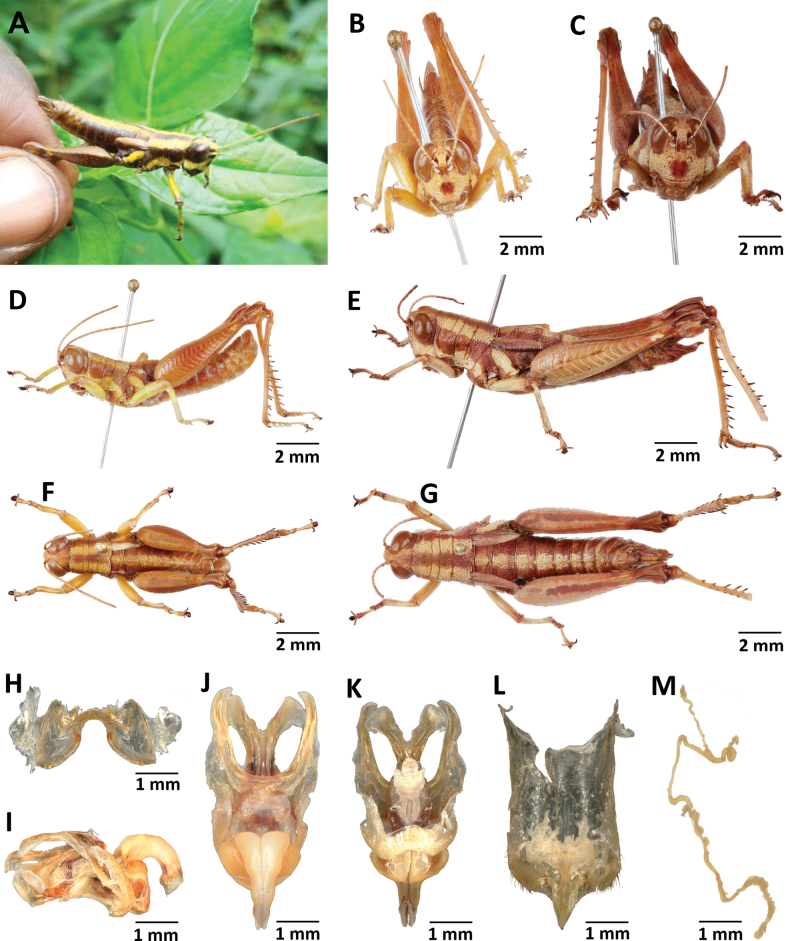
*Pteroperamatzkei* sp. nov. **A** image of a female under natural conditions **B** male frontal view **C** female frontal view **D** male lateral view **E** female lateral view **F** male dorsal view **G** female dorsal view **H** epiphallus dorsal view **I** phallic complex lateral view **J** phallic complex dorsal view **K** phallic complex ventral view **L** female subgenital plate **M** female spermatheca.

**Male**: Body and legs with inconspicuous hairs; integument moderately rugous dorsally, and smooth ventrally; general coloration dark brown with yellow bands; eyes of medium size; antennae thin, filiform, longer than head and pronotum together, with 21 segments; median subocellar facial spot single or unique; longitudinal median dark band on pronotum disc as wide as adjacent yellow bands, but widened behind the typical groove; basal yellow bands of lateral lobes of pronotum narrowed in front of the second transverse groove, but not interrupted; median carina distinct, crossed dorsolaterally by three sulci; lateral carinae absent; prozona longer than metazona; prosternal process short conical or pyramidal; mesosternal interspace open; meso- and metathoracic episternites entirely yellow; tegmina lobiform, narrow, brown in the lower half and yellow in the upper half, covering the tympanum; wings less developed; fore and middle legs entirely yellow, more or less pale; hind femur almost entirely dark brown, with a pregenicular yellow spot on the external and inner areas; dorsal carinae of hind femora with slight tooth; hind tibiae dark yellow, basal ring absent; external apical spines of the hind tibiae absent; spines on the hind tibiae varying from seven to eight in both external and internal sides; male subgenital plate acute in dorsal view; the pallium and supra-anal plate of male is not raised; the male cerci long, conical, incurved and exceeding beyond the supra-anal plate. ***Epiphallus*** (Fig. 13H): large; bridge narrow, arched; ancorae small, strongly curved, interiorly directed; lateral plates domed, extending back from the bridge; anterior projections triangular. ***Phallic complex*** (Fig. 13I–K): Dorsal arch of cingulum closed, U-shaped, its apical 2/3 overlapping with endophallic sclerites; apodemes of the cingulum incurved and extending beyond endophallic apodemes; rami slightly bent; lateroventral sclerites narrow, as high as long; aedeagus of larger size, curved, forming a quarter circle; membranous apex of aedeagus, outside endophallic sheaths, supported by a longitudinal division of upper valve, short, broad; apex of aedeagus with a ridge-like expansions; membranous apex of aedeagus subtriangular, downcurved in lateral view; upper ectophallic sheath not enlarged at its base and tightly molding the aedeagus valves; lower ectophallic sheath broad, capping the base of rami.

**Female**: As male but larger; cerci short conical; subgenital plate (Fig. 13L) pentagonal, broad, with rounded posterior margins; anterior apodemes thin, acute; egg-guide broad with acute apex; distal recurrent trunk of lateral spermathecal diverticulum 3× longer than proximal trunk; spermathecal ampulla elongate; base of spermathecal duct narrow (Fig. 13M).

##### Measurements.

Males (mm) (*n* = 4): total length of body 20.57–21.79; length of pronotum 4.17–4.33; length of hind femur 13.48–13.56; length of elytra 3.90–4.63. Females (mm) (*n* = 2): total length of body 27.25–28.05; length of pronotum 5.39–5.55; length of hind femur 15.91–16.89; length of elytra 4.95–6.10; length of ovipositor 2.96–3.30. Additional measurement information is shown in Table 1.

##### Etymology.

The species was named after Mr. Danilo Matzke, an important taxonomist for Dermaptera in Germany for his dedication and scientific contributions to the taxonomy of earwigs.

##### Habitat.

Dense evergreen forest in the Congo Basin, in the forest along the Dja River.

##### Distribution.

At present, the species is known only from Somalomo in the Dja Biosphere Reserve, Cameroon (Fig. 17B).

#### ﻿ Pteropera missoupi

Taxon classificationAnimaliaOrthopteraAcrididae

Yetchom & Husemann sp. nov.

DD10F6FF-E785-5F38-9D73-F99DB44CF487

https://zoobank.org/8DD173E3-68BE-40BE-B5A1-54684A435581

Fig. 14A–M

##### Materials examined.

***Holotype*.** Cameroon • ♂; Iboti, in the Ebo forest; 4°27.001'N, 10°27.002'E, 731 m a.s.l.; 7 Jan. 2022; J.A. Yetchom Fondjo leg.; SMNK, SMNK-ORTH-0000003. ***Paratypes***. Cameroon • 1 ♂; Ongot; 3°51.517'N, 11°22.367'E; 20 March 2020; J.A. Yetchom Fondjo leg.; SMNK. Cameroon • 5 ♂♂, 3 ♀♀; Ongot; 3°51.517'N, 11°22.367'E; 15 Jun. 2020; J.A. Yetchom Fondjo & A.R. Nzoko Fiemapong leg.; SMNK). Cameroon • 1 ♂, 1 ♀; Ongot; 3°51.517'N, 11°22.367'E; 5 Dec. 2021; J.A. Yetchom Fondjo leg.; SMNK. Cameroon. • 2 ♂♂, 3 ♀♀; Iboti, in the Ebo forest; 4°27.001'N, 10°27.002'E, 731 m a.s.l.; 7 Jan. 2022; J.A. Yetchom Fondjo leg.; SMNK.

##### Diagnosis.

*Pteroperamissoupi* sp. nov. differs from *Pteroperabalachowskyi* Donskoff, 1981 (Figs 3C, D, 8C, D) in the following features: subocellar facial spot fused, single (divided in *P.balachowskyi*); dorsal and ventral areas of abdomen yellowish or greenish (brownish in *P.balachowskyi*); male cerci with a short inner lobe (male cerci simple in *P.balachowskyi*); male genitalia differ in its U-shaped and close dorsal arch of the cingulum (strongly open in *P.balachowskyi*); upper ectophallic sheath short, not bent (bent and globular in *P.balachowskyi*).

The new species is similar to *Pteroperajeanninae* Donskoff, 1981 (Figs 4E, F, 9C, D) from which it differs in that its subocellar facial spot is single (divided into two in *P.jeanninae*); its meso- and metathoracic episternites are pale (yellowish, conspicuous in *P.jeanninae*); its hind femur has a pale green internal area, with a yellowish median band extending towards the lower margin (uniformly dark green in *P.jeanninae*); male genitalia differ in its U-shaped dorsal arch of the cingulum (V-shaped in *P.jeanninae*); and the upper ectophallic sheath is short, not bent (elongate in *P.jeanninae*).

The new species is also similar to *Pteroperacarnapi* Ramme, 1929 (Figs 3A, B, 8A, B), from which it differs in the following characteristics: meso- and metathoracic episternites are pale (yellow in the middle in *P.carnapi*); hind tibia dark green (green bluish in *P.carnapi*); inner lobe of male cerci shorter than the outer one (as long as or longer than the outer lobe in *P.carnapi*); and male genitalia differ in its U-shaped, close dorsal arch of cingulum enveloping, apodemes reaching the apex of endophallic sclerites (open forward, apodemes not exceeding the point of separation of endophallic valves in *P.carnapi*).

The new species *Pteroperamissoupi* sp. nov. is similar to *Pteroperamirei* Donskoff, 1981 (Figs 5C, D, 10A, B) from Cameroon in terms of coloration and the shape of the male cercus. However, it can be distinguished from *P.mirei* by the following characteristics: median subocellar facial spot single not divided in both sexes, but divided in males, sometimes confluent in females of *P.mirei*; basal pale bands on the lateral lobes of the pronotum narrowed and not interrupted in front of the second transverse groove, but interrupted in *P.mirei*; and male genitalia differ in the following characteristics: in *Pteroperamissoupi* sp. nov., the two membranous tips at the end of the aedeagus belong to the upper valve, whereas in *P.mirei*, each of the two membranous tips at the end of the aedeagus belongs to a separate valve. In addition, in *Pteroperamissoupi* sp. nov., the end of the lower tip caps the upper tip, whereas the end of the upper tip caps the lower tip in *P.mirei*.

##### Description.

**Figure 14.**
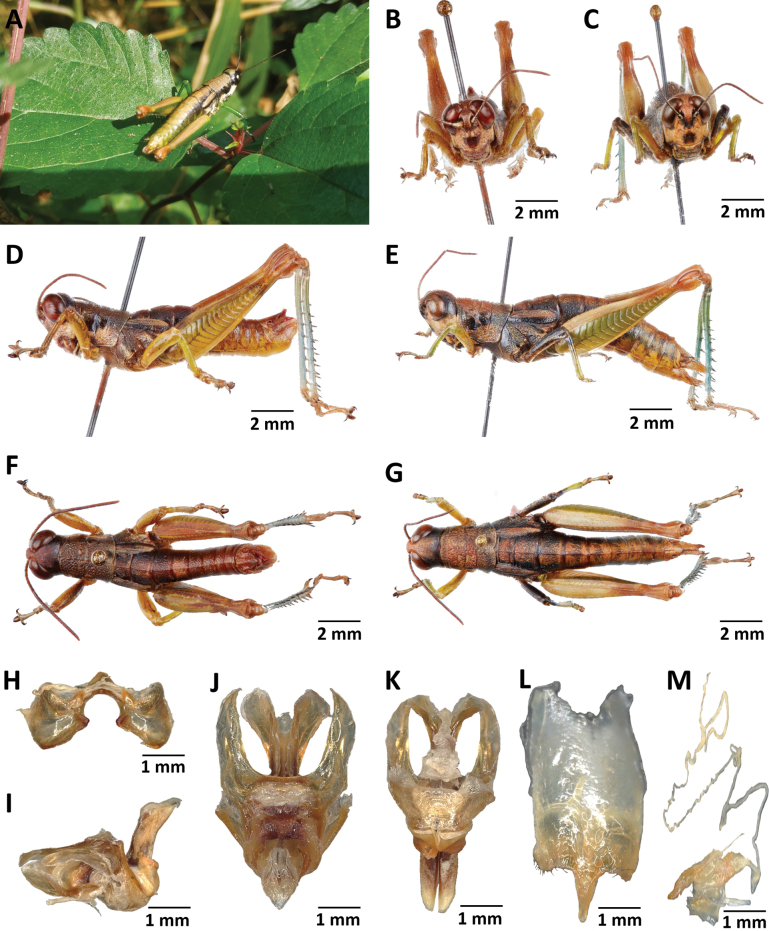
*Pteroperamissoupi* sp. nov. **A** habitus image of a female under natural conditions **B** male frontal view **C** female frontal view **D** male lateral view **E** female lateral view **F** male dorsal view **G** female dorsal view **H** epiphallus dorsal view **I** phallic complex lateral view **J** phallic complex dorsal view **K** phallic complex ventral view **L** female subgenital plate **M** female spermatheca.

**Male**: Body and legs with inconspicuous hairs; integument moderately rugous dorsally and smooth ventrally; eyes prominent; antennae thin, filiform, longer than head and pronotum together; large subocellar facial spot fused in a single spot; vertex, dorsal area of pronotum, external upper area of hind femur pale brown; dark longitudinal median band on pronotum disc absent; basal pale band on lateral lobes of pronotum narrowed in front of the second transverse sulcus but not interrupted; prozona longer than metazona; prosternal process conical in its apical part; meso- and metathoracic episternites pale; tegmina lobiform, only slightly reaching the third abdominal segment; mesosternal interspace open; dorsal and ventral area of abdomen yellowish; fore- and middle legs, external and upper inner areas of hind femur dark green; median and lower inner areas of hind femur yellow; knee brownish; hind tibiae dark blue in fresh specimens; male cerci with a short inner lobe; subgenital plate obtusely rounded in dorsal view; pallium and supra-anal plate of male raised. **Epiphallus** (Fig. 14H): oval sclerites smaller; ancorae smaller, incurved; lateral margins divergent. **Phallic complex** (Fig. 14I–K): dorsal arch of the cingulum U-shaped, close, apodemes only reaching the apex of the endophallic sclerites but not exceeding them; rami bent at an obtuous angle; lateroventral sclerites triangular; zygoma reduced; upper aedeagus valve not bent; upper ectophallic sheath short, not bent; lower ectophallic sheath of smaller size, enveloping the base of the rami; aedeagus short, curved; free end of the aedeagus, outside the ectophallic sheaths, bifid, with broad-sized tips; two membranous tips at the end of the aedeagus belonging to the upper valve; and the end of the lower tip capping the upper tip.

**Female**: As male but larger; cerci short conical; subgenital plate (Fig. 14L) subrectangular, with straight anterior margins; anterior apodemes narrow and short; egg-guide thin and long; the recurrent distal trunk of lateral spermathecal diverticulum almost 2.5× longer than the proximal trunk; spermathecal ampulla narrowed at apex; spermathecal duct very long; the base of the copulatory bursa at least as far from the basivalvar sclerites arc as the distance between them; copulatory bursa straight; the base of the spermathecal duct slightly enlarged; copulatory bursa tapering to mid-height; angle formed by the two basivalvar sclerites rounded (Fig. 14M).

##### Measurements.

Males (mm) (*n* = 8): total length of body 18.97–22.91; length of pronotum 3.95–4.74; length of hind femur 12.65–13.77; length of elytra 3.11–4.27. Females (mm) (*n* = 7): total length of body 24.41–28.70; length of pronotum 5.14–5.62; length of hind femur 15.43–17.36; length of elytra 4.32–5.40; length of ovipositor 2.28–3.75. Additional measurement information is shown in Table 1.

##### Etymology.

The species is dedicated to Prof. Alain Didier Missoup in recognition of his work and achievements in the systematic and evolutionary biology of small mammals in Cameroon.

**Figure 15.**
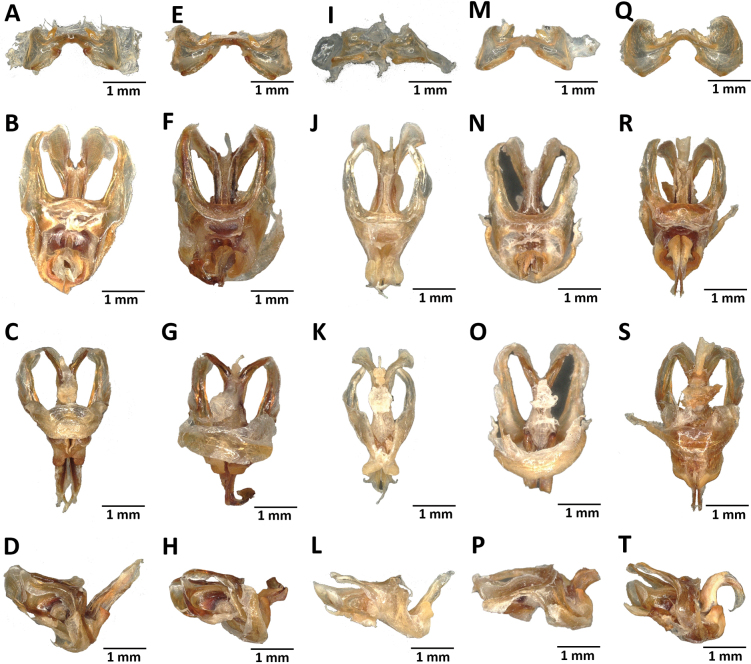
Internal genitalia of *P.carnapi* (**A–D**), *P.descampsi* (**E–H**), *P.karschizenkeri* (**I–L**), *P.uniformis* (**M–P**) and *P.verrucigena* (**O–T**).

**Figure 16.**
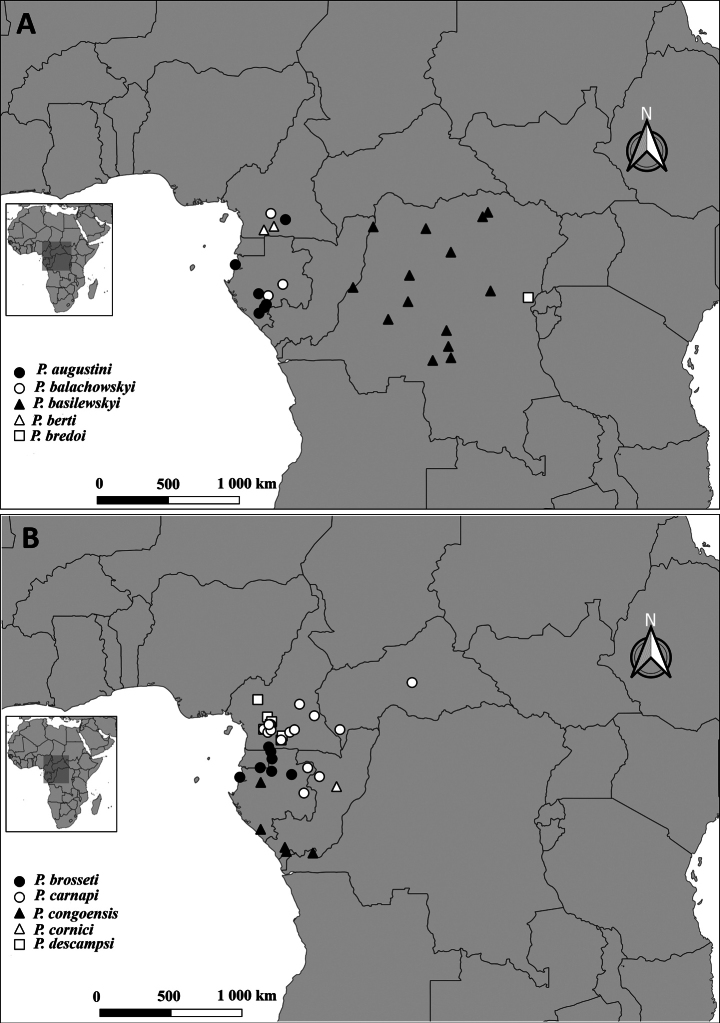
Distribution of *Pteropera* species in African rainforests.

##### Habitat.

Dense evergreen forests in the Ebo forest; degraded forests and along the forest edges.

##### Distribution.

Iboti in the Ebo forest; Ongot in the Centre region, Cameroon (Fig. 18A).

**Figure 17.**
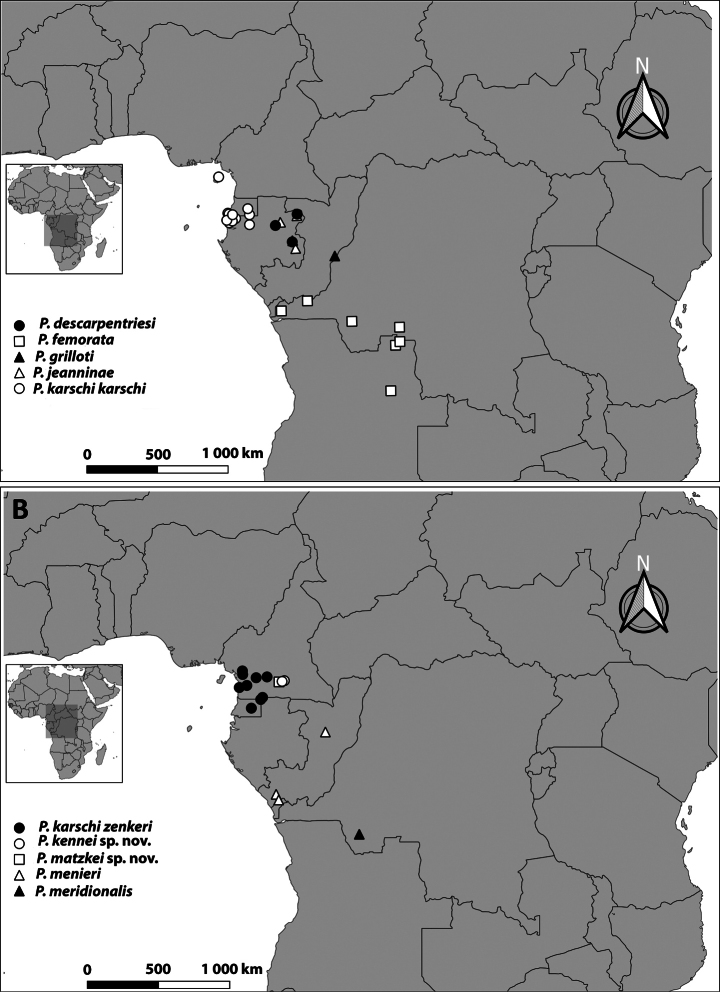
Distribution of *Pteropera* species in African rainforests.

**Figure 18.**
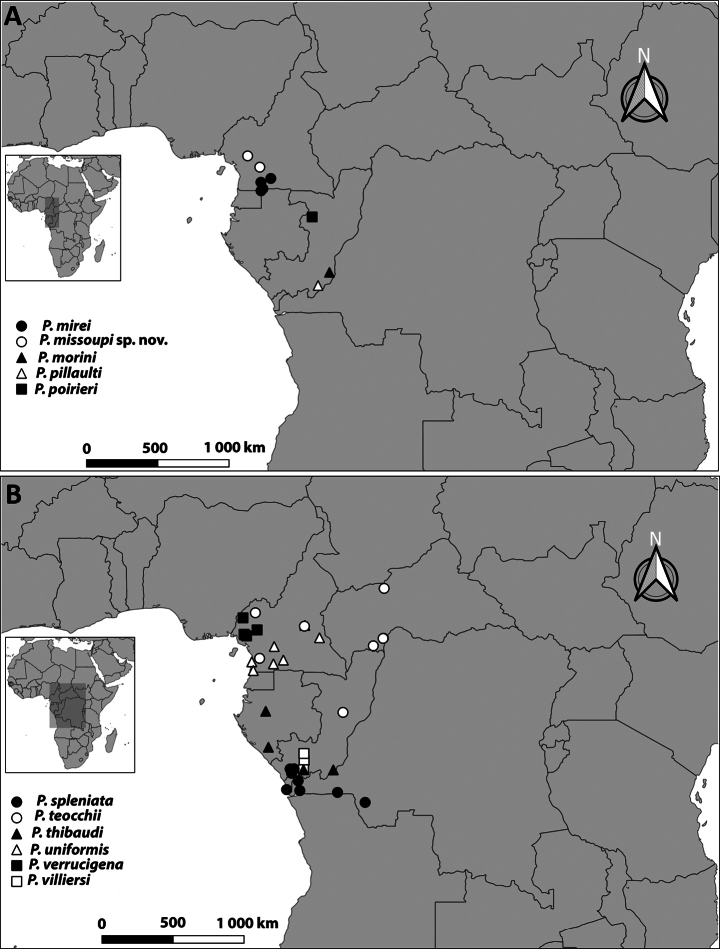
Distribution of *Pteropera* species in African rainforests.

### ﻿Updated keys to species of *Pteropera*

The keys provided here are derived from those presented by Donskoff (1981) but have been altered to include the newly described species of the genus *Pteropera*.

#### ﻿A. Based on external morphology of males and females

**Table:** 

1	Three pale spots on the outer area of hind femora (Fig. 2A, B, G–I)	**2**
–	No pale spots on the outer hind femora	**13**
2	A dark longitudinal median band on pronotum disc (Fig. 13F, G)	**3**
–	No dark longitudinal median band on pronotal disc (Fig. 2G, H, 7G, H)	***P.brosseti* Donskoff, 1981** [Equatorial Guinea, Gabon]
3	A pale median spot on the inner side of the hind femora in the upper or central position	**4**
–	No pale median spot on the inner side of the hind femora; beginning of spots along the upper edge, near the outer spots	**7**
4	Base of the inner area of the hind femora red or orange. Hind tibiae red or orange	**5**
–	Base of the inner side of the hind femora dark green or reddish brown. Hind tibiae black or dark green	**6**
5	Male cerci extending beyond the end of the supra-anal plate (Fig. 4G, I)	***P.karschi* (I. Bolívar, 1905)** [Cameroon, Equatorial Guinea, Gabon, Fernando Poo]
–	Male cerci not extending beyond the end of the supra-anal plate. Hind tibiae always red (Fig. 6E)	***P.thibaudi* Donskoff, 1981** [Congo-Brazzaville, Gabon]
6	The pale basal band on the lateral lobes of the pronotum extending from the anterior edge to the posterior edge (Fig. 2A, B)	***P.augustini* Donskoff, 1981** [Cameroon, Gabon]
–	The pale basal band of the lateral lobes of the pronotum limited to the posterior half (Fig. 2I)	***P.basilewskyi* Donskoff, 1981** [Democratic Republic of Congo]
7	The pale basal band on the lateral lobes of the pronotum narrows before the second transverse groove but not interrupted	**8**
–	The pale basal band of the lateral lobes of the pronotum interrupted in front of the second transverse groove by at least a thin dark line	**11**
8	Male cerci without inner preapical lobule	**9**
–	Male cerci with inner preapical lobule (Fig. 5G)	***P.pillaulti* Donskoff, 1981** [Congo-Brazzaville]
9	Hind tibiae black or wine-red; lower-external areas of hind femora darkened	**10**
–	Hind tibiae green; infero-external areas of hind femora pale (Fig. 3I, J)	***P.descarpentriesi* Donskoff, 1981** [Gabon]
10	Hind tibiae wine-red (Fig. 6G, H)	***P.verrucigena* Karsch, 1891** [Cameroon]
–	Hind tibiae black (Fig. 6C, D)	***P.teocchii* Donskoff, 1981** [Cameroon, Central African Republic, Congo-Brazzaville]
11	Male cerci thickened or very slightly bifid (Fig. 4A)	***P.femorata* (Giglio-tos, 1907)** [Angola, Congo-Brazzaville, Democratic Republic of Congo]
–	Male cerci acute	**12**
12	Male cerci with inner preapical lobule (Fig. 3C)	***P.congoensis* Donskoff, 1981** [Congo-Brazzaville, Democratic Republic of Congo, Gabon]
–	Male cerci without inner preapical lobule (Fig. 5A, 9I)	***P.menieri* Donskoff, 1981** [Congo-Brazzaville]
13	Hind tibiae more or less dark yellow (Fig. 13D–G)	***P.matzkei* sp. nov.** [Cameroon]
–	Hind tibiae more or less dark green	**14**
14	A dark longitudinal median band on pronotum disc	**15**
–	No dark longitudinal median band on the pronotum disc	**23**
15	A dark longitudinal median band on pronotum disc twice as wide as each of the adjoining pale bands (Fig. 9B)	***P.meridionalis* Donskoff, 1981** [Democratic Republic of Congo]
–	A dark longitudinal median band on the pronotum disc as wide as each of the adjoining pale bands	**16**
16	Inner area of the hind femora almost entirely red without pale spots (Fig. 4C, 9A)	***P.grilloti* Donskoff, 1981** [Congo-Brazzaville]
–	Inner side of hind femora more or less dark green	**17**
17	The pale basal band on the lateral lobe of the pronotum completely absent (Fig. 12A, D, E)	***P.kennei* sp. nov.** [Cameroon]
–	The pale basal band on the lateral lobes of pronotum present	**18**
18	The pale basal band of the lateral lobes of pronotum interrupted at the level of the second transverse groove by at least a thin dark line	**19**
–	The pale basal band of the lateral lobes of pronotum not interrupted at the second transverse groove	**21**
19	A large V-shaped facial spot extending from cheek to cheek; male cerci short	**20**
–	A large subocellar facial spot, dark, median, sometimes divided into two small symmetrical spots centered on the carinae of the frontal side	***P.spleniata*** (Karsch, 1896) [Congo-Brazzaville, Democratic Republic of Congo]
20	Male subgenital plate rounded (in dorsal view) (Fig. 11I)	***P.uniformis* Bruner, 1920** [Cameroon]
–	Male subgenital plate truncated (in dorsal view) (Fig. 8G)	***P.descampsi* Donskoff, 1981** [Cameroon]
21	Male cerci acute (Fig. 6I, 7E, 11G)	**22**
–	Male cerci bilobed, subocellar facial patch separated into two (Fig. 3E)	***P.cornici* Donskoff, 1981** [Congo-Brazzaville]
22	A large subocellar facial spot single, median	***P.bertii* Donskoff, 1981** [Cameroon]
–	A large subocellar facial spot divided into two small symmetrical spots centered on the carinae of the front side	***P.villiersi* Donskoff, 1981** [Congo-Brazzaville]
23	Basal half of the inner part of hind femora red; hind tibiae red at the base, green at the apex (Fig. 2J)	***P.bredoi* Donskoff, 1981** [Democratic Republic of Congo]
–	Inner part of hind femora green	**24**
24	The pale basal band of the lateral lobes of the pronotum always interrupted at the second transverse groove (Fig. 5C, D)	***P.mirei* Donskoff, 1981** [Cameroon, Gabon]
–	The pale basal band of the lateral lobes of the pronotum generally not interrupted at the second transverse groove	**25**
25	The pale basal band on the lateral lobes of the pronotum widened in front of the second transverse groove	**26**
–	The pale basal band of the lateral lobes of the pronotum narrow in front of the second transverse groove	**27**
26	The pale basal band on the lateral lobes of the pronotum very wide and extends to the lower edge of these lateral lobes. Male cerci with preapical inner lobule (Fig. 5E, F)	***P.morini* Donskoff, 1981** [Congo-Brazzaville]
–	Pale basal band on the lateral lobes of pronotum, narrow, with parallel edges. Male cerci tapered (Fig. 5I, J)	***P.poirieri* Donskoff, 1981** [Congo-Brazzaville]
27	Male cerci simple; pallium and supra-anal plate not raised (Fig. 2C)	***P.balachowskyi* Donskoff, 1981** [Cameroon, Gabon]
–	Male cerci bilobed; pallium and supra-anal plate raised	**28**
28	Inner lobe of male cerci as long as or longer than the outer lobe (Fig. 8A)	***P.carnapi* Ramme, 1929** [Cameroon, Central African Republic, Congo-Brazzaville, Gabon]
–	Inner lobe of male cerci shorter than the outer lobe (Fig. 4E)	**29**
29	A lage subocellar facial spot divided into two	***P.jeanninae* Donskoff, 1981** [Gabon]
–	A large subocellar facial spot fused (Fig. 14B, C)	***P.missoupi* sp. nov.** [Cameroon]

#### ﻿B. Based on the male internal genitalia

**Table:** 

1	End of the aedeagus, outside the ectophallic sheaths, bifid, pointed (Fig. 14I–K)	**2**
–	End of aedeagus simple	**7**
2	The two membranous tips at the end of aedeagus belonging to the upper valve	**3**
–	Each of the two membranous tips at the end of the aedeagus belonging to a different valve	**5**
3	The two tips of the end of the aedeagus widened, the end of the lower tip caps the upper tip (Fig. 14I–K)	***P.missoupi* sp. nov.** [Cameroon]
–	The two tips at the end of the aedeagus, thin	**4**
4	The two tips at the end of the aedeagus curved downwards	***P.balachowskyi* Donskoff, 1981** [Cameroon, Gabon]
–	The lower tip at the end of the aedeagus thin, and the upper tip widened and semicircular, both in line with the valve	***P.villiersi* Donskoff, 1981** [Congo-Brazzaville]
5	Aedeagus very long, straight, upper tip filiform, lower tip lamellar, lanceolate	***P.jeanninae* Donskoff, 1981** [Gabon]
–	Aedeagus shorter, curved	**6**
6	The upper ectophalic sheath extended. End of the upper valve of the aedeagus caps the lower valve	***P.mirei* Donskoff, 1981** [Cameroon, Gabon]
–	Upper ectophallic sheath globular. Tips of both valves convergent, plier-like	***P.brosseti* Donskoff, 1981** [Equatorial Guinea, Gabon]
7	Membranous apex of aedeagus, outside the sheaths, supported by two sclerites, formed by the longitudinal division of the upper valve	**8**
–	Membranous apex of the aedeagus, outside the sheaths, without sclerites	**10**
8	Aedeagus strongly curved, upper ectophalic sheath widened dorsally at the base	***P.descarpentriesi* Donskoff, 1981** [Gabon]
–	Aedeagus slightly curved, upper ectophallic sheath not extended at the base (Fig. 13I, J)	**9**
9	Dorsal arch of cingulum slightly open, not reaching apex of endophallic valves nor overhanging them apically	***P.bertii* Donskoff, 1981** [Cameroon]
–	Dorsal arch of cingulum closed, long, extending beyond the apex of endophallic valves, and overhanging them apically (Fig. 13J, K)	***P.matzkei* sp. nov.** [Cameroon]
10	Aedeagus almost straight	**11**
–	Aedeagus curved	**15**
11	Aedeagus large, upper ectophallic sheath cylindrical, long, membranous apex of upper valve thin, acute (Fig. 15B–D)	***P.carnapi* Ramme, 1929** [Cameroon, Central African Republic, Congo-Brazzaville, Gabon]
–	Aedeagus small, Upper ectophallic sheath globular at the apex	**12**
12	Membranous apex of the upper valve of aedeagus horizontal or oblique	**13**
–	Membranous apex of the upper valve of aedeagus truncate	**14**
13	Membranous apex of the upper valve of aedeagus lamellar, horizontal, in the extension of the valve, oval, acute	***P.karschikarschi* (I. Bolívar, 1905)** [Equatorial Guinea, Gabon, Fernando Poo]
–	Membranous apex of the upper valve of aedeagus lamellar, curved upward, oblique in lateral view (Fig. 15 J–L)	***P.karschizenkeri* Ramme, 1929** [Cameroon, Equatorial Guinea]
14	Lower ectophallic sheath enveloping, lateral	***P.augustini* Donskoff, 1981** [Cameroon, Gabon]
–	Lower ectophallic sheath nonenveloping, posterior	***P.thibaudi* Donskoff, 1981** [Congo-Brazzaville, Gabon]
15	Aedeagus curved upwards	**16**
–	Aedeagus curved downwards	**17**
16	Membranous apex of the aedeagus in diverging pallets (Fig. 15 F–H)	***P.descampsi* Donskoff, 1981** [Cameroon]
–	Membranous apex of the aedeagus in converging hooks	***P.uniformis* Bruner, 1920** [Cameroon]
17	Aedeagus small, slightly curved; membranous apex in short triangular blade	***P.basilewskyi* Donskoff, 1981** [Democratic Republic of Congo]
–	Aedeagus large, well-curved	**18**
18	Membranous apex of the aedeagus long, filiform	**19**
–	Membranous apex of the aedeagus never filiform	**23**
19	Lower ectophallic sheath, small, nonenveloping	**20**
–	Lower ectophallic sheath, large, enveloping	**21**
20	Upper aedeagus valve thin, regularly curved	***P.femorata* (Giglio-tos, 1907)** [Angola, Congo-Brazzaville, Democratic Republic of Congo]
–	Upper aedeagus valve widened into a transverse blade (Fig. 12I–K)	***P.kennei* sp. nov.** [Cameroon]
21	Upper ectophallic sheath long, slightly curved; membranous apex of aedeagus recurrent	***P.menieri* Donskoff, 1981** [Congo-Brazzaville]
–	Upper ectophallic sheath short, strongly curved; membranous apex of aedeagus extending to the curvature of the upper valve	**22**
22	Base of upper ectophallic sheath molding the valves of the aedeagus; membranous apex of the aedeagus long and thin	***P.spleniata* (Karsch, 1896)** [Congo-Brazzaville, Democratic Republic of Congo]
–	Base of upper ectophallic sheath swollen dorsally; membranous apex of aedeagus shorter, hook-like	***P.congoensis* Donskoff, 1981** [Congo-Brazzaville, Democratic Republic of Congo, Gabon]
23	Apex of the aedeagus with a ridge-like expansion	***P.cornici* Donskoff, 1981** [Congo-Brazzaville]
–	Apex of aedeagus without ridge-like expansion	**24**
24	Apex of aedeage short, rounded	***P.poirieri* Donskoff, 1981** [Congo-Brazzaville]
–	Apex of aedeagus acute or widened	**25**
25	Apex of aedeagus widened	**26**
–	Apex of aedeagus acute	**27**
26	Apex of aedeagus widened into a rounded spatula	***P.pillaulti* Donskoff, 1981** [Congo-Brazzaville]
–	Apex of aedeagus widened into a transverse angular, self-wrapped blade	***P.grilloti* Donskoff, 1981** [Congo-Brazzaville]
27	Aedeagus strongly curved, curvature accentuated by the molded ectophallic sheath, long tip	**28**
–	Aedeagus slightly curved, ectophallic sheath widened dorsally at the base, tip short	***P.morini* Donskoff, 1981** [Congo-Brazzaville]
28	Aedeagus forming only a semicircle (Fig. 15T)	***P.verrucigena* Karsch, 1891** [Cameroon]
–	Aedeagus forming an almost complete circle	***P.teocchii*** Donskoff, 1981 [Cameroon, Central African Republic, Congo-Brazzaville]

#### ﻿C. Based on female internal genitalia

**Table:** 

1	Bottom of the copulatory bursa at least as far from the arc of the basivalvar sclerites as their spacing	**2**
–	Bottom of the copulatory bursa close to the arc of the basivalvar sclerites	**6**
2	The base of spermathecal duct widened to a length equal to the distance between the basivalvar sclerites and parallel to the copulatory bursa	**3**
–	Copulatory bursa curved	**5**
3	Copulatory bursa with parallel edges. Angle formed by the two basivalvar sclerites obtuse	***P.jeanninae* Donskoff, 1981** [Gabon]
–	Copulatory bursa tapering to mid-height	**4**
4	Angle formed by the two basivalvar sclerites right	***P.carnapi* Ramme, 1929** [Cameroon, Central African Republic, Congo-Brazzaville, Gabon]
–	Angle formed by the two basivalvar sclerites rounded (Fig. 14M)	***P.missoupi* sp. nov.** [Cameroon]
5	Copulatory bursa above the basivalvar sclerites, formed by a thick ventral gutter and a membranous roof. The base of the spermathecal duct widened, very short, hooked. Basivalvar sclerites bent, obtuse	***P.brosseti* Donskoff, 1981** [Equatorial Guinea, Gabon]
–	Bottom of copulatory bursa thickened, regularly narrowed. The base of the spermathecal duct widened over a large distance and coiled into two inverted spiral arcs. Basivalvar sclerites not bent, almost straight	***P.mirei* Donskoff, 1981** [Cameroon, Gabon]
6	Copulatory bursa almost straight	**7**
–	Copulatory bursa arch-shaped	**11**
7	The base of the spermathecal duct opening laterally into the bursa. Roof of the bursa membranous	***P.villiersi* Donskoff, 1981** [Congo-Brazzaville]
–	Base of the spermathecal duct leading to the apex of the copulatory bursa	**8**
8	Each basivalvar sclerite straight or only slightly curved	**9**
–	Each basivalvar sclerite angular	***P.basilewskyi* Donskoff, 1981** [Democratic Republic of Congo]
9	Copulatory bursa without internal sclerite	***P.karschi* (I. Bolívar, 1905)** [Cameroon, Equatorial Guinea, Gabon, Fernando Poo]
–	Copulatory bursa with two internal sclerites	**10**
10	The two inner sclerites of the copulatory bursa rounded	***P.augustini* Donskoff, 1981** [Cameroon, Gabon]
–	The two inner sclerites of the copulatory bursa elongate	***P.thibaudi* Donskoff, 1981** [Congo-Brazzaville, Gabon]
11	Distal, recurrent section of the lateral spermathecal diverticulum 5× longer than the proximal section	**12**
–	Distal, recurrent section of the lateral spermathecal diverticulum < 5× as long as the proximal section	**13**
12	Spermathecal duct fine, widening into a small ampulla at the outlet into the copulatory bursa	***P.poirieri* Donskoff, 1981** [Congo-Brazzaville]
–	Spermathecal duct gradually widening at the base	***P.menieri* Donskoff, 1981** [Congo-Brazzaville]
13	Arch formed by the copulatory bursa short, medial sclerite, single	**14**
–	Arch of copulatory bursa long, well-curved or wrapped	**18**
14	Inner sclerite of the copulatory bursa fine	**15**
–	Inner sclerite of the copulatory bursa broad and narrow at the apex. Angle formed by the two basivalvar sclerites obtuse	**16**
15	Angle formed by the two basivalvar sclerites acute	***P.uniformis* Bruner, 1920** [Cameroon]
–	Angle formed by the two basivalvar sclerites obtuse (Fig. 12M)	***P.kennei* sp. nov.** [Cameroon]
16	Inner sclerite of copulatory bursa short. Distal recurrent section of the lateral diverticulum of the spermatheca 4× longer than the proximal section	***P.descarpentriesi* Donskoff, 1981** [Gabon]
–	Inner sclerite of the copulatory bursa long. Distal recurrent section of the lateral diverticulum of the spermatheca 3× as long as the proximal section	**17**
17	Spermathecal ampulla broad at the apex	***P.bertii* Donskoff, 1981** [Cameroon]
–	Spermathecal ampulla elongated at the apex (Fig. 13M)	***P.matzkei* sp. nov.** [Cameroon]
18	Arch of copulatory bursa wrapped, forming more than one complete turn. Internal median sclerite of copulatory bursa acute at both ends	***P.cornici* Donskoff, 1981** [Congo-Brazzaville]
–	Arch of copulatory bursa less wrapped, forming less than one turn	**19**
19	Arch of copulatory bursa describing a half-turn	**20**
–	Arch of the copulatory bursa describing only a quarter turn	**27**
20	Copulatory bursa gradually narrow at the apex	**21**
–	Copulatory bursa abruptly narrow at the apex	**22**
21	Apex of the copulatory bursa descending at the joint of the basivalvar sclerites	***P.verrucigena* Karsch, 1891** [Cameroon]
–	Apex of the bursa descending below the joint of the basivalvar sclerites	***P.teocchii* Donskoff, 1981** [Cameroon, Central African Republic, Congo-Brazzaville]
22	Basivalvar sclerites almost straight	**23**
–	Basivalvar sclerites angular	**24**
23	Basivalvar sclerites acute	***P.descampsi* Donskoff, 1981** [Cameroon]
–	Basivalvar sclerites obtuse	***P.meridionalis* Donskoff, 1981** [Democratic Republic of Congo]
24	Basivalvar sclerites forming a right angle. Arch of copulatory bursa thin and slender	***P.femorata* (Giglio-tos, 1907)** [Angola, Congo-Brazzaville, Democratic Republic of Congo]
–	Basivalvar sclerites obtuse, arch of copulatory bursa short	**25**
25	Base of the spermathecal duct widened	***P.bredoi* Donskoff, 1981** [Democratic Republic of Congo]
–	Base of spermathecal duct narrow	**26**
26	Anterior apodemes of the subgenital plate short	***P.spleniata* (Karsch, 1896)** [Congo-Brazzaville, Democratic Republic of Congo]
–	Anterior apodemes of the subgenital plate long	***P.congoensis* Donskoff, 1981** [Congo-Brazzaville, Democratic Republic of Congo, Gabon]
27	Roof of the copulatory bursa membranous. The base of the spermathecal duct widened to twice the length of the space between the bases of the two basivalvar sclerites	***P.balachowskyi* Donskoff, 1981** [Cameroon, Gabon]
–	Bottom of the copulatory bursa thickened. The base of the spermathecal duct widened forming a short arch	**28**
28	Distal, recurrent section of the lateral spermathecal diverticulum > 4× longer than the proximal section, well-enveloping	***P.pillaulti* Donskoff, 1981** [Congo-Brazzaville]
–	Distal, recurrent section of the lateral diverticulum of the spermatheca 3× longer than the proximal section, less enveloping	***P.morini* Donskoff, 1981** [Congo-Brazzaville]

## ﻿Discussion

Although some attempts have been made to generate the DNA barcode data of orthopterans and mantids from the Central African Republic, Gabon, Ivory Coast, and South Africa (Moulin et al. 2017; Massa et al. 2018; O’Hare et al. 2023), no molecular attempts focusing on Orthoptera have been made in Cameroon thus far. Thus, the present study presents the first barcode data of morphologically identified *Pteropera* species from Cameroon. Many species of this diversified genus are not included in our analyses, as most of them are known only from museum collections of old samples for which it was not possible to extract DNA. We included only Cameroonian species for which we had fresh samples in the trees. Our results revealed that all these *Pteropera* species were monophyletic, including the newly described taxa. Hence, with the combined data, we are confident that the newly discovered taxa are indeed valid species. Hence, our study demonstrates the potential of using classic DNA barcoding to delimit species and the use of a multilocus dataset to estimate well-supported phylogenetic trees. However, further studies including a larger dataset are needed to obtain a more complete image of the true diversity of the genus.

The works by Ramme (1929) and Donskoff (1981) have thus far been the only contributions to the taxonomy of the genus *Pteropera*. Most species of this genus have restricted distributions; 21 of the 27 previously known species have been recorded only from single localities. In this study, we review the genus through an integrated taxonomic approach and describe three new species. We highlight some morphological differences in some species in comparison with Donskoff’s (1981) descriptions. In *P.carnapi* for example, the apodemes of the cingulum were slender and exceeded the level of separation of endophallic valves, with a strongly incurved apex. In contrast, these structures do not exceed the level of separation of the endophallic valves according to Donskoff (1981). In addition, Donskoff’s (1981) descriptions of *P.descampsi* revealed that the apodemes of the cingulum as project at the level of the ejaculatory sac, whereas these structures reach the apex of the endophallic sclerites according to our observations. These differences observed in both studies related to *P.carnapi* and *P.descampsi* could be explained by the intraspecific variation that may have occurred within the genus *Pteropera*.

Moreover, we failed to find three *Pteropera* species previously reported from Cameroon, probably because we did not have the opportunity to sample the localities where they were reported and because most species are narrow endemics. These species are *P.bertii and P.mirei* (both known from Koemvone and Ebolowa in the southern part of Cameroon) and *P.teocchii* (known from Bafut in the western part and Goyoum in the eastern part of Cameroon).

Nevertheless, the distribution range of *P.augustini*, which is known only from Gabon, was extended in this study, as we were able to report the species from Cameroon for the first time. The new record of *P.augustini*, combined with the description of three new species, increases the number of *Pteropera* present in Cameroon from eight to 12 species, and overall to 30 species and subspecies that are currently recorded from Afrotropical regions. Nevertheless, this genus may be more diverse than currently known, given the large number of localities in the African rainforests that have not yet been investigated in general and in Cameroon in particular. Thus, further sampling efforts at different locations and habitat types are needed.

## Supplementary Material

XML Treatment for Pteropera

XML Treatment for Pteropera augustini

XML Treatment for Pteropera carnapi

XML Treatment for Pteropera descampsi

XML Treatment for Pteropera karschi zenkeri

XML Treatment for Pteropera uniformis

XML Treatment for Pteropera verrucigena

XML Treatment for Pteropera kennei

XML Treatment for Pteropera matzkei

XML Treatment for Pteropera missoupi

## ﻿Additional information

### Author ORCIDs

Jeanne Agrippine Yetchom Fondjo https://orcid.org/0000-0003-2192-6366

Armand Richard Nzoko Fiemapong https://orcid.org/0000-0002-8759-8142

Maurice Tindo https://orcid.org/0000-0001-6217-6982

Tarekegn Fite Duressa https://orcid.org/0000-0003-3492-4933

Slobodan Ivković https://orcid.org/0000-0001-5571-8245

Martin Husemann https://orcid.org/0000-0001-5536-6681

### Data availability

All of the data that support the findings of this study are available in the main text or Supplementary Information.

## Supplementary materials

Supplementary material 1 Locations and coordinates of all species of *Pteropera* Data type csv Explanation note Localities and geographical coordinates used to map the distribution of *Pteropera* species (CAR = Central African REPUBLIC; DRC = Democratic Republic of Congo). https://binary.pensoft.net/file/1161007This dataset is made available under the Open Database License (http://opendatacommons.org/licenses/odbl/1.0/). The Open Database License (ODbL) is a license agreement intended to allow users to freely share, modify, and use this Dataset while maintaining this same freedom for others, provided that the original source and author(s) are credited.Jeanne Agrippine Yetchom Fondjo, Armand Richard Nzoko Fiemapong, Maurice Tindo, Tarekegn Fite Duressa, Slobodan Ivković, Martin Husemann
